# Correction: Reversal of the adipostat control of torpor during migration in hummingbirds

**DOI:** 10.7554/eLife.86017

**Published:** 2023-01-11

**Authors:** Erich R Eberts, Christopher G Guglielmo, Kenneth C Welch

**Keywords:** Other

Eberts ER, Guglielmo CG, Welch Jr KC. 2021. Reversal of the adipostat control of torpor during migration in hummingbirds. *eLife* **10**:e70062. doi: 10.7554/eLife.70062.

Published 6 December 2022

We recently noticed three mistakes in the original published manuscript, regarding (1) how a certain subset of values was reported, (2) phrasing of a methodological point, and (3) a figure citation. These errors arose by accidental oversight of changes late in the review process. Details of these errors and how they have been corrected are as follows:

1. We intended to reported amount of fat as milligrams of fat, but mistakenly reported the values as centigrams of fat, so the original values were 1 order of magnitude off from the units reported. We have updated these values to reflect the proper units of mg fat. No statistical relationships change (though a few p-values very slightly change and have been updated). These changes have been updated in the main text as well as the supplementary file.

2. In the methods, we mistakenly specified that "We defined ‘arousal’ as periods where the rate of change of was greater than *four* standard deviations higher than the mean rate of change during the *normothermic period before torpor*;" but the text should, and now does, instead read: “We defined ‘arousal’ as periods where the rate of change was greater than *five* times the standard deviation of the mean rate of change during the *torpid* period.”

3. In the results section, we incorrectly cited Figure 2A where we intended to cite Figure 2B: "and lost more fat mass overnight (slope: 1.00±0.17 mgfat%fat–1, *P*<0.001; Figure 2A)." This citation has now been corrected to read “Figure 2B”.

The article has been corrected accordingly.

