# Supplementary material for: Reversal of the adipostat control of torpor during migration in hummingbirds
Source: eLife. 2021 Dec 6;10:e70062. doi: 10.7554/eLife.70062 (PMC8719877; doi:10.7554/eLife.70062)
Supplement: Supplementary file 1. — (A) Estimated marginal means and slopes of body composition and food consumption variables, descriptions of the statistical models, and statistical comparisons within and among periods. (B) Absolute and relative increases in body mass, fat mass, and fat content during the fattening period. (C) Correlations between body mass gains and duration of the fattening period, with mean torpor duration in the fattening period and food consumption in the fattening period. (D) Estimated marginal means and slopes of torpor use variables, descriptions of the statistical models, and statistical comparisons within and among periods. [file elife-70062-supp1.docx]

**Supplementary Tables**

**Supplementary File 1A.** Estimated marginal means and slopes of body composition and food consumption variables, descriptions of the statistical models, and statistical comparisons within and among periods.

|  | **Body Mass (morning)** | | | **Fat Content (fat mass/body mass)** | | | |  |
| --- | --- | --- | --- | --- | --- | --- | --- | --- |
| **Model ^a^** | ~ Period | ~ Period *  Date | | | ~ Period | ~ Period *  Date | | |
| **Period (n)** | **Mean ± SE (g)** | **Slope ± SE (g/day)** | ***p*** | | **Mean ± SE (%)** | **Slope ± SE**  **(%/day)** | ***p*** | |
| Breeding  (13) | 2.77±0.05  (F, M) ^b^ | 0.00±0.00  (F) | 0.338 | | 4.70±0.80  (F, M) | 0.04±0.02  (F) | 0.053 | |
| Fattening (13) | 3.31± 0.06  (B, M) | 0.02 ± 0.00* ^c^  (B, M) | <0.001 | | 19.60±1.14 (B, M) | 0.81± 0.12*  (B, M) | <0.001 | |
| Migration (13) | 3.73± 0.05  (B, M) | 0.00±0.00  (F) | 0.079 | | 29.90±0.83 (B, F) | 0.11±0.06  (F) | 0.061 | |
| Non-fatteners (3) | 2.81±0.13 | 0.00±0.00 | 0.201 | | 7.69±3.28 | 0.15±0.02 | <0.001 | |

**Supplementary File 1A. (cont.)** Estimated marginal means and slopes of body composition and food consumption variables, descriptions of the statistical models, and statistical comparisons within and among periods.

|  | **Lean Mass** | | | **Food Consumption** | | | | |  |
| --- | --- | --- | --- | --- | --- | --- | --- | --- | --- |
| **Model ^a^** | ~ Period | ~ Period *  Date | | ~ Period + Day Length | ~ Period * Week +  Period * Day Length | | | |  |
| **Period (n)** | **Mean ± SE (g)** | **Slope ± SE (g/day)** | ***p*** | **Mean ± SE (kJ/day)** | **Slope ± SE (kJ/day)** | ***p*** | **Slope ± SE (kJ/h_day_)** | ***p*** | |
| Breeding  (13) | 2.67±0.03  (M) | 0.00±0.00*  (M) | 0.020 | 67.4±2.2 (M) | 6.2±0.9*  (M) | <0.001 | 27.0±4.13 | <0.001 | |
| Fattening (13) | 2.80±0.05  (M) | 0.00±0.01  (--) | 0.848 | 66.0±2.7  (M) | -6.7±2.5*  (--) | 0.008 | -9.1±5.8 | 0.111 | |
| Migration (13) | 2.55±0.03 (B, F) | -0.02±0.00*  (B) | <0.001 | 57.6±2.8  (B, F) | -2.7±1.8  (B) | 0.114 | 8.7±4.8 | 0.068 | |
| Non-fatteners (3) | 2.52±0.04 | 0.00±0.00* | 0.004 | 69.5±7.8 | 3.6±1.5* | 0.015 | 17.4±5.1* | 0.001 | |
|  |  |  |  | Daylength:  *p=*0.009;  Square root transformation of Food Consumption |  |  |  |  | |

^a^ Asterix in model indicates interaction.

^b^ Statistically significant comparisons within parentheses, alpha taken at 0.05 (P<0.05).

^c^ Asterix after value indicates significance.

**Supplementary File 1B.** Absolute and relative increases in body mass, fat mass, and fat content during the fattening period.

|  | **Absolute** | | **Relative (%)** | |
| --- | --- | --- | --- | --- |
| **Value** | **Mean ± SE** | **Range** | **Mean ± SE** | **Range** |
| Fat Duration (days) | 10±1 | 6-18 | -------- | -------- |
| Body Mass Increase (g) | 0.58±0.05 | 0.34-0.90 | 19.6±1.6 | 11.6, 29.0 |
| Fat Mass Increase (g) | 0.60±0.05 | 0.34-0.84 | 216.2±28.8 | 83.1, 486.1 |
| Fat Content Increase (%) | 15.0±0.9 | 8.8-19.1 | 163.7±23.4 | 63.7, 390.1 |

**Supplementary File 1C.** Correlations between body mass gains and duration of the fattening period, with mean torpor duration in the fattening period and food consumption in the fattening period.

|  | **Body Mass Gains (g)** | | **Fat Duration (days)** | |
| --- | --- | --- | --- | --- |
| **Model** | Body Mass Gains (g) ~  Mean Torpor Duration (h) +  Mean Food Intake (kJ) | | Fat Duration (days) ~  Mean Torpor Duration (h) +  Mean Food Intake (kJ) | |
|  | **Slope ± SE** | ***p*** | **Slope ± SE** | ***p*** |
| **Mean Torpor Duration (h)** | 0.07±0.01* ^a^ | 0.004 | -0.60±0.75 | 0.468 |
| **Mean Weekly Food Intake (mL/day)** | 0.00±0.00 | 0.996 | -0.27±0.25 | 0.343 |

^a^ Asterix after value indicates significance.

**Supplementary File 1D**. Estimated marginal means and slopes of torpor use variables, descriptions of the statistical models, and statistical comparisons within and among periods.

|  | **Torpor Propensity** | | |  |  | **Duration** | | | | | |
| --- | --- | --- | --- | --- | --- | --- | --- | --- | --- | --- | --- |
| **Model** ^a^ | ~ Period | ~ Period * Evening Fat % | | ~ Period * Night Length | | ~ Period + Night Length | ~ Period * Evening Fat % +  Period * Night Length | | | |  |
| **Period (n)** | **Mean ± SE (%)** | **Slope ± SE** | ***p*** | **Slope ± SE** | ***p*** | **Mean ± SE (h_torpor_)** | **Slope ± SE**  **(h_torpor_ / %_fat_)** | ***p*** | **Slope ± SE**  **(h_torpor_ / h_night_)** | ***p*** |  |
| Breeding  (13) | 61.6±11.1 | -0.70±0.22* ^c^  (F, M) ^b^ | 0.001 | -0.31±0.47 | 0.452 | 4.31±0.56 | -0.63±0.10*  (F, M) | <0.001 | 0.52±0.32 | 0.077 |  |
| Fattening  (13) | 40.1±11.8 | 0.24±0.16  (B) | 0.132 | 1.02±1.43 | 0.476 | 3.61±0.81 | -0.07±0.12  (B) | 0.533 | 3.07±1.07 * | 0.003 |  |
| Migration (13) | 65.1±11.1 | 0.26±0.11*  (B) | 0.017 | 2.06±0.94* | 0.028 | 3.68±0.47 | 0.10±0.07  (B) | 0.124 | 1.18±0.58 * | 0.031 |  |
| Non-fatteners (3) | 48.6±10.1 | 0.06±0.06 | 0.376 | 0.01±0.04 | 0.831 | 2.95±0.49 | -0.09±0.23 | 0.181 | 0.67±1.30 | 0.180 |  |
|  | No pairwise differences:  *p*>0.101 |  |  |  |  | No pairwise differences:  *p*>0.743  Night Length: *p*=0.761 |  |  | No pairwise differences:  *p*>0.060 |  |  |

**Supplementary File 1D**. **(cont.)** Estimated marginal means and slopes of torpor use variables, descriptions of the statistical models, and statistical comparisons within and among periods.

|  | **Pre-Torpor Energy Expenditure** | | | | | | | **Time of Torpor Entry** | | | | | |
| --- | --- | --- | --- | --- | --- | --- | --- | --- | --- | --- | --- | --- | --- |
| **Model ^a^** | ~ Period +  Night Length | ~ Period * Evening Fat % +  Period * Night Length | | | | | ~ Period + Night Length | | ~ Period * Evening Fat % +  Period * Night Length | | | |  |
| **Period (n)** | **Mean ± SE**  **(kJ)** | **Slope ± SE (kJ/ %_fat_)** | ***p*** | **Slope ± SE (kJ/ h_night_)** | ***p*** | | **Mean ± SE**  **(h_entry_)** | | **Slope ± SE**  **(h_entry_/ %_fat_)** | ***p*** | **Slope ± SE**  **(h_entry_/ h_night_)** | ***p*** |  |
| Breeding  (13) | 3.19±0.37 | 0.39±0.06*  (F, M) | <0.001 | 0.40±0.22*  (F) | | 0.041 | 4.67±0.53 | | 0.55±0.09*  (F, M) | <0.001 | 0.52±0.30  (F) | 0.054 |  |
| Fattening (13) | 3.73±0.55 | 0.05±0.09  (B) | 0.528 | -2.48±0.75*  (B, M) | | <0.001 | 5.24±0.77 | | 0.05±0.12  (B) | 0.630 | -3.02±1.03 *  (B) | 0.002 |  |
| Migration (13) | 4.04±0.31 | -0.06±0.05  (B) | 0.204 | -0.41±0.41  (F) | | 0.276 | 5.53±0.45 | | -0.08±0.07  (B) | 0.230 | -0.51±0.56  (--) | 0.333 |  |
| Non-fatteners (3) | 4.10±0.32 | 0.07±0.15 | 0.091 | 0.05±0.81 | | 0.883 | 6.16±0.41 | | -0.02±0.18 | 0.769 | 0.44±0.92 | 0.315 |  |
|  | No pairwise differences:  *p* >0.307;  Night Length:  *p*=0.015 |  |  |  | |  | No pairwise differences:  *p* >0.538  Night Length: p=0.012 | |  |  |  |  |  |

^a^ Asterix in model indicates interaction.

^b^ Statistically significant comparisons within parentheses, alpha taken at 0.05 (P<0.05).

^c^ Asterix after value indicates significance.

**Supplementary File 1D**. **(cont.)** Estimated marginal means and slopes of torpor use variables, descriptions of the statistical models, and statistical comparisons within and among periods.

| **Fat % at Torpor Entry** | | | | | | | | |
| --- | --- | --- | --- | --- | --- | --- | --- | --- |
| **Model** | ~ Period | ~ Period | ~ Period * Time of Entry +  Period* Night Length | | | | ~Period *  Date | |
| **Period (n)** | **Mean ± SE**  **(%_fat_)** | **Mean ± SE**  **(mg_fat_)** | **Slope ± SE (%_fat_/ h_entry_)** | ***p*** | **Slope ± SE (%_fat_/ h_night_)** | ***p*** | **Slope± SE (%_fat_/ Date)** | ***p*** |
| Breeding  (13) | 5.56±0.79  (F, M) | 142.84±39.17  (F, M) | 0.38±0.31  (M) | 0.191 | 0.84±0.78 | 0.253 | 0.05±0.02 * | 0.042 |
| Fattening  (13) | 26.72±1.51  (B, M) | 932.12±72.27  (B, M) | 3.72±1.45 *  (--) | 0.007 | 14.25±5.71 | 0.009 | 0.14±0.24 | 0.538 |
| Migration  (13) | 32.94±0.77  (B, F) | 1249.08±38.34  (B, F) | -1.20±0.35*  (B) | <0.001 | 0.99±1.41 | 0.450 | -0.06±0.07 | 0.402 |
| Non-fatteners  (3) | 10.34±5.33 | 314.37±180.05 | 0.30±0.52 | 0.54 | 3.33±0.75 | <0.001 | 0.14±0.03 | <0.001 |
|  | Night length does not improve model AIC | Night length does not improve model AIC |  |  | No pairwise differences:  *p* >0.057 |  | No pairwise differences:  *p* >0.921  Night length does not improve model AIC |  |

**Supplementary File 1D**. **(cont.)** Estimated marginal means and slopes of torpor use variables, descriptions of the statistical models, and statistical comparisons within and among periods.

|  | **Torpid Overnight Fat Mass Loss** | | | | | | |
| --- | --- | --- | --- | --- | --- | --- | --- |
| **Model ^a^** | ~ Period + Night Length | ~ Period * Evening Fat % +  Period* Night Length | | | | ~ Period * Torpor Duration +  Night Length | |
| **Period (n)** | **Mean ± SE**  **(mg_fat_)** | **Slope ± SE**  **(mg_fat_/ %_fat_)** | ***p*** | **Slope ± SE**  **(mg _fat_/ h_night_)** | ***p*** | **Slope ± SE (mg_fat_/ %_fat_)** | ***p*** |
| Breeding  (13) | 123.32±9.23 | 9.98±1.75*  (F, M) | <0.001 | 13.52±5.97 *  (F) | 0.014 | -17.23±0.52*  (M) | <0.001 |
| Fattening  (13) | 142.61±13.42 | 1.78±2.34  (B) | 0.403 | -48.25±20.02 *  (B) | 0.010 | -16.82±1.02* | <0.001 |
| Migration  (13) | 144.36±7.70 | -1.54±1.24  (B) | 0.148 | -0.258±10.83  (--) | 0.979 | -15.35±0.72*  (B) | <0.001 |
| Non-fatteners  (3) | 144.05±15.61 | 3.55±3.88* | 0.003 | -1.34±21.53 | 0.868 | -16.47±1.04* | <0.001 |
|  | No pairwise differences: *p* >0.307;  Night Length: *p*<0.001 |  |  |  |  | Night Length: *p*<0.001 |  |

^a^ Asterix in model indicates interaction.

^b^ Statistically significant comparisons within parentheses, alpha taken at 0.05 (P<0.05).

^c^ Asterix after value indicates significance.
