# Supplementary material for: Reversal of the adipostat control of torpor during migration in hummingbirds
Source: eLife. 2021 Dec 6;10:e70062. doi: 10.7554/eLife.70062 (PMC8719877; doi:10.7554/eLife.70062)
Supplement: Supplementary file 2. — Red lines represent normothermic nights and blue lines represent torpid nights. The average breeding threshold ±1 standard error is indicated by horizontal dashed black and greay lines, respectively. [file elife-70062-supp2.pdf]

Supplementary File 2. Instantaneous percent fat content over time (as % of night) throughout each summer, fattening, and migration period, as well as during the whole study period for non-fatteners. Red lines represent normothermic nights and blue lines represent torpid nights. The average breeding threshold ± 1 standard error is indicated by horizontal dashed black and grey lines, respectively.

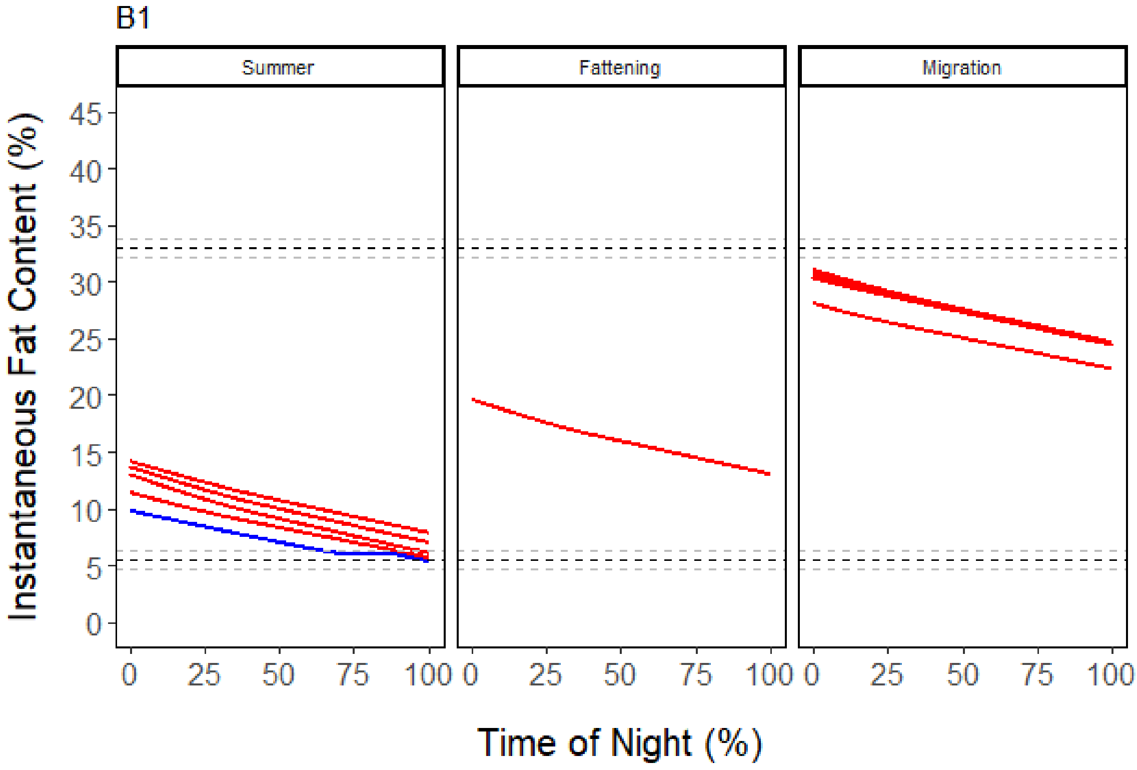

B2

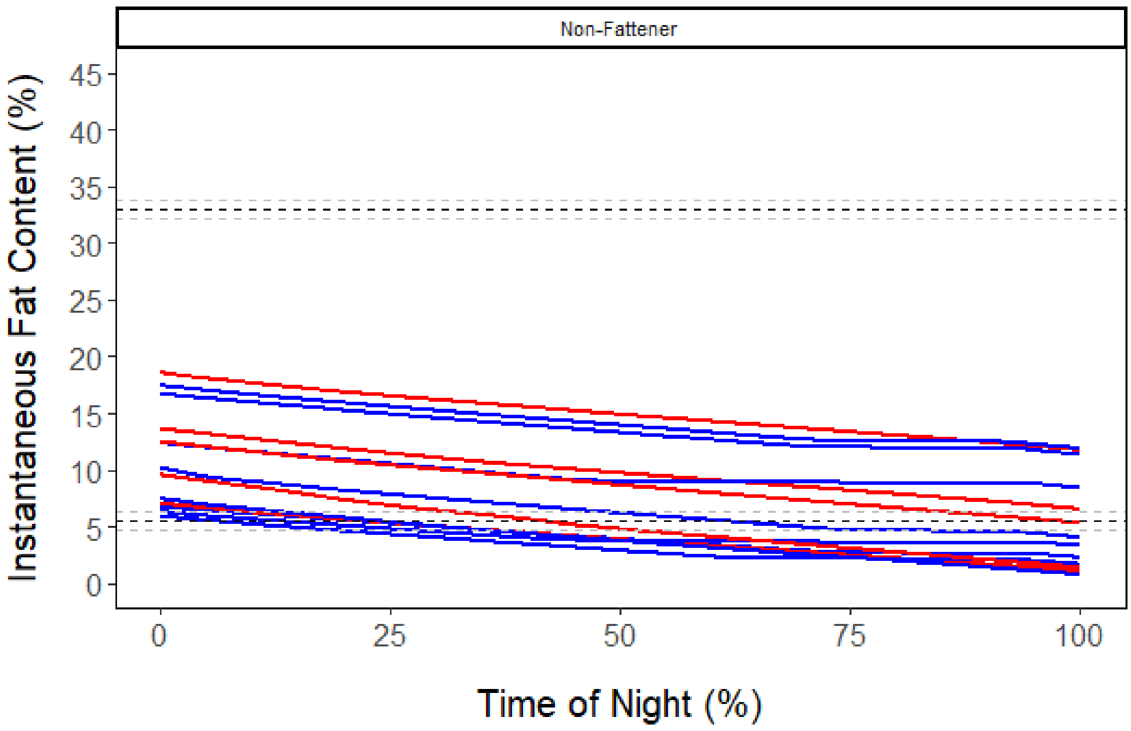
Non-Fattener
Instantaneous Fat Content (%)
45
40
35
30
25
20
15
10
5
0
0
25
50
75
100
Time of Night (%)

B3

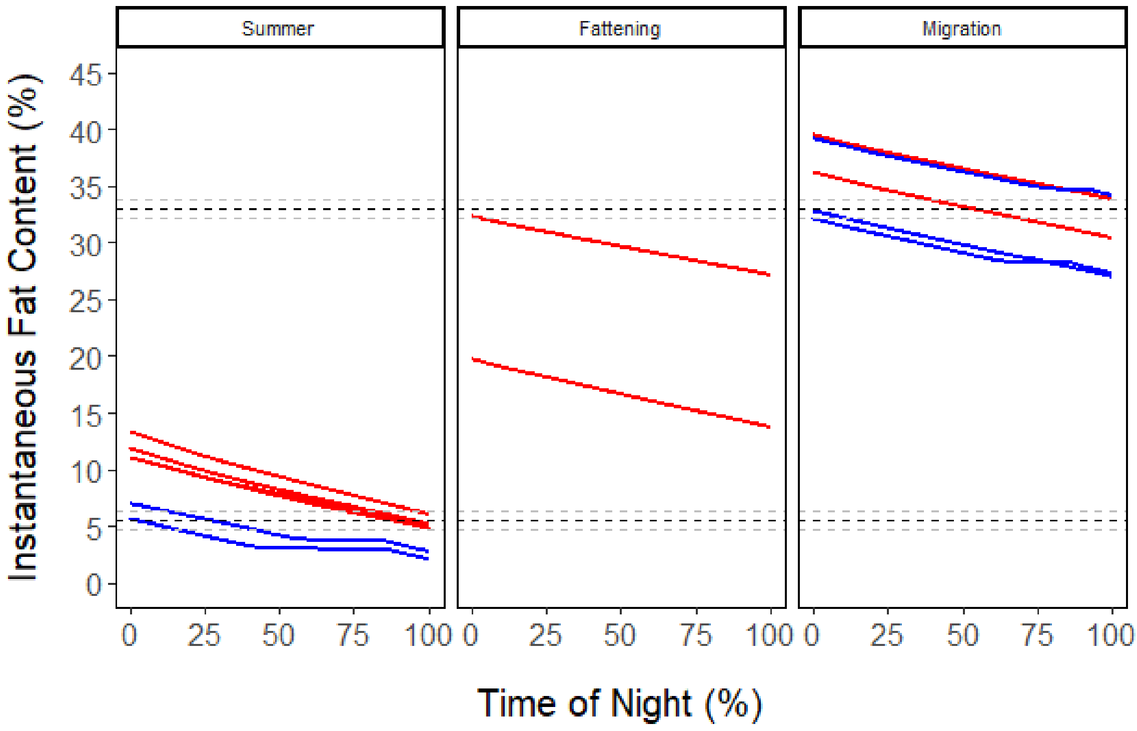
Summer
Fattening
Migration
Instantaneous Fat Content (%)
45
40
35
30
25
20
15
10
5
0
0
25
50
75
100
Time of Night (%)

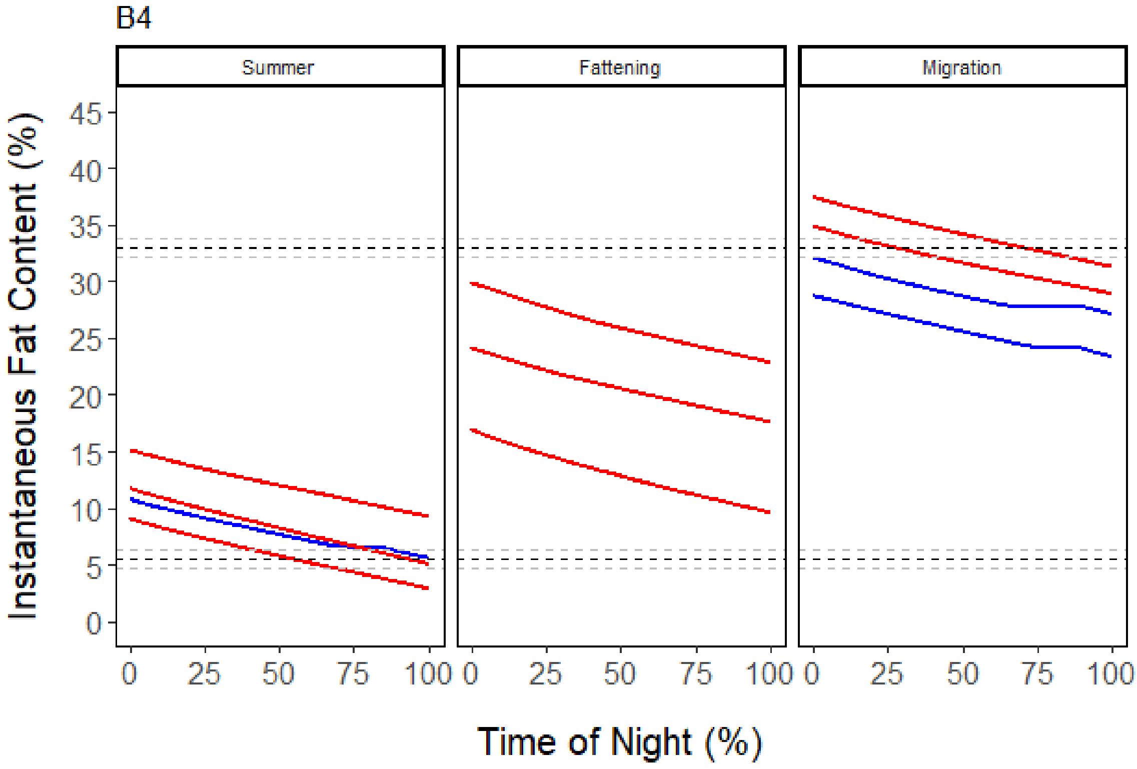
B4
Summer
Fattening
Migration
Instantaneous Fat Content (%)
45
40
35
30
25
20
15
10
5
0
0
25
50
75
100
Time of Night (%)

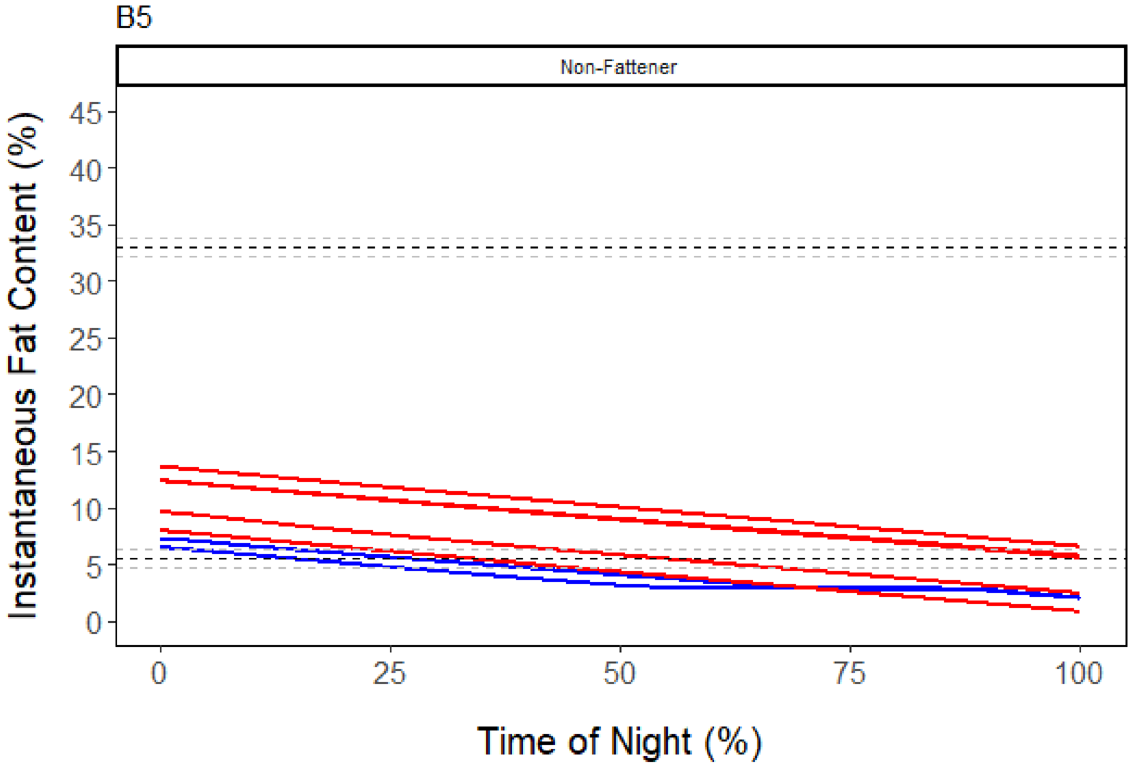
B5
Non-Fattener
Instantaneous Fat Content (%)
45
40
35
30
25
20
15
10
5
0
0
25
50
75
100
Time of Night (%)

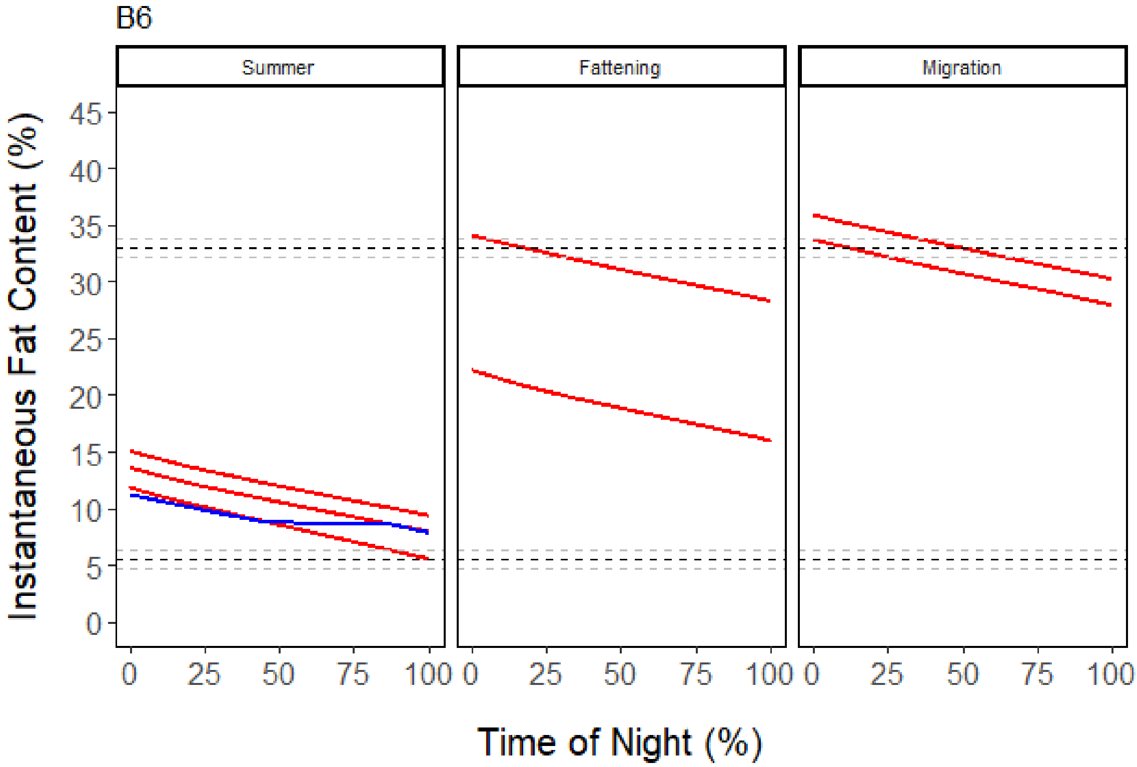
B6
Summer
Fattening
Migration
Instantaneous Fat Content (%)
45
40
35
30
25
20
15
10
5
0
0
25
50
75
100
Time of Night (%)

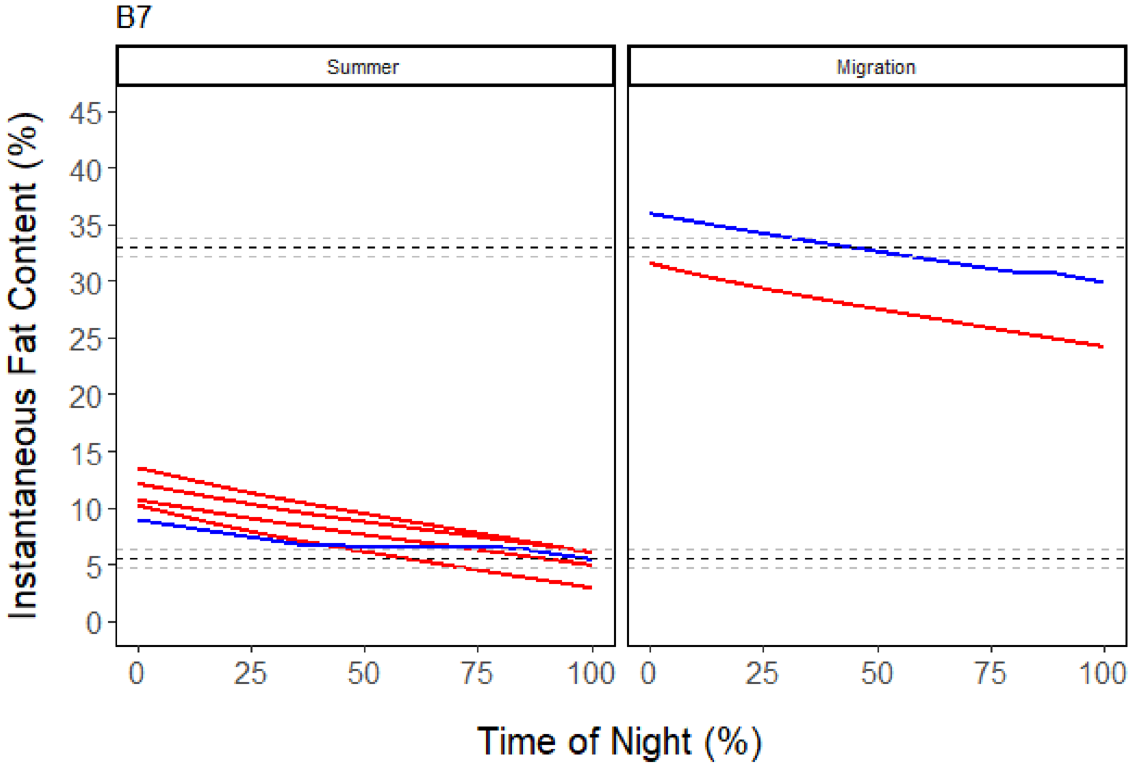
B7
Summer
Migration
Instantaneous Fat Content (%)
45
40
35
30
25
20
15
10
5
0
0
25
50
75
100
Time of Night (%)

B8

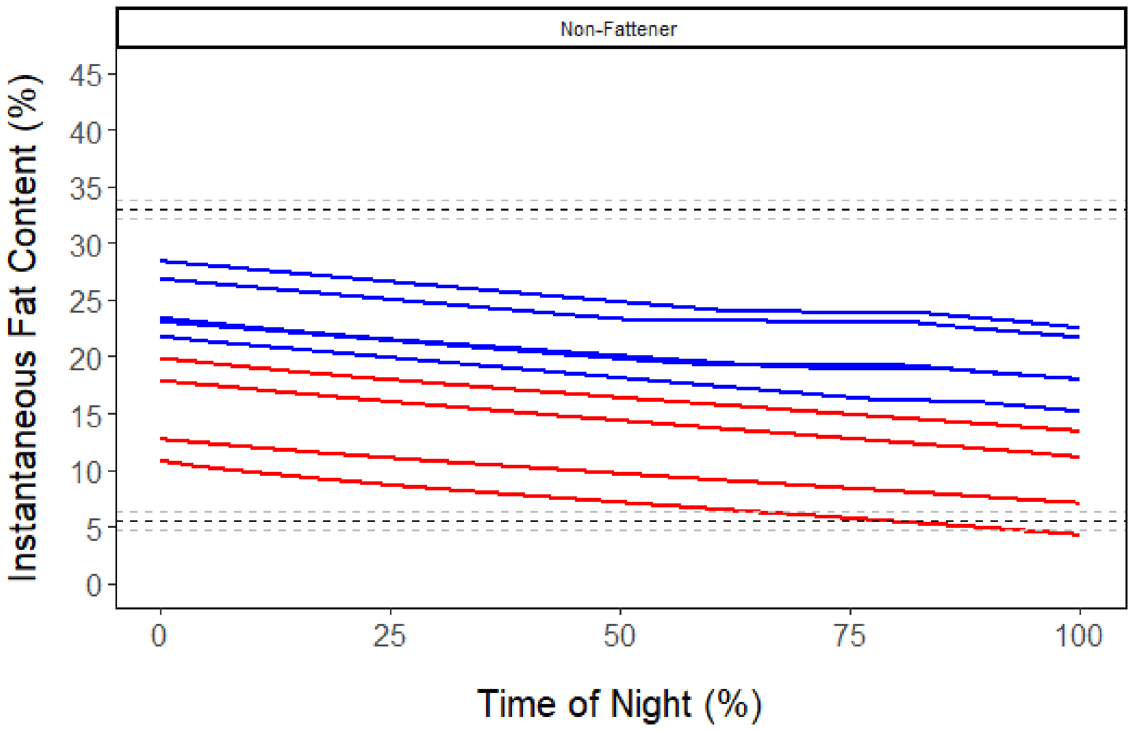
Non-Fattener
Instantaneous Fat Content (%)
45
40
35
30
25
20
15
10
5
0
0
25
50
75
100
Time of Night (%)

B9

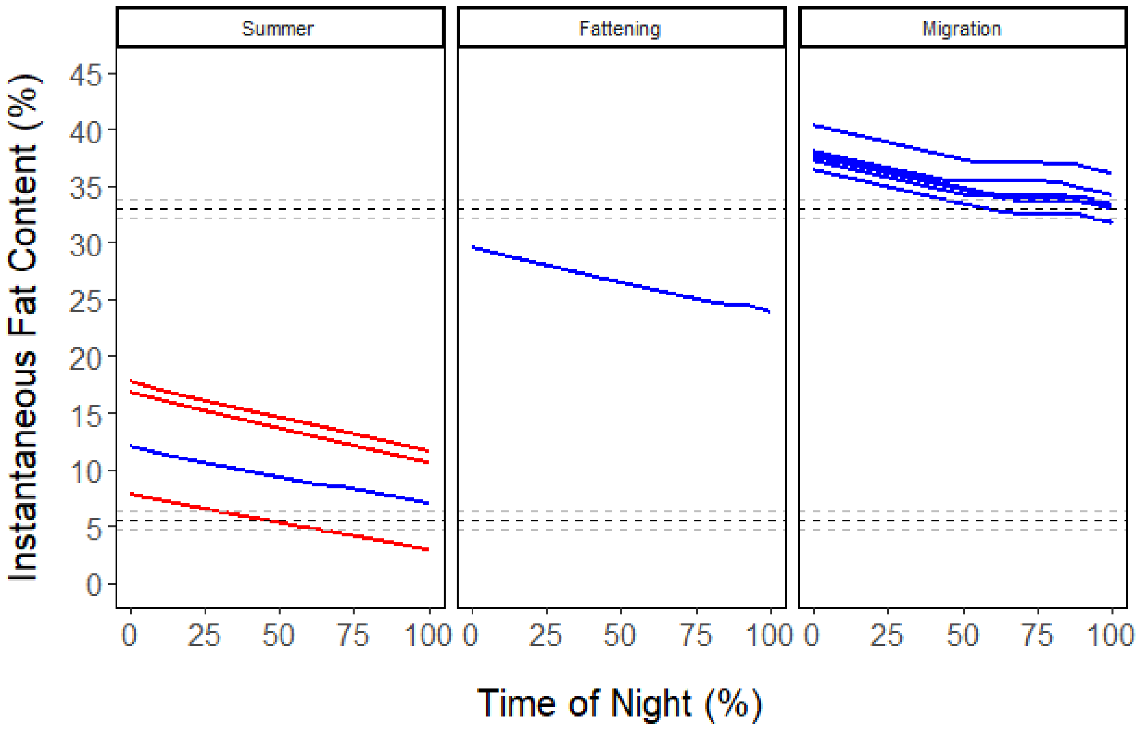
Summer
Fattening
Migration
Instantaneous Fat Content (%)
45
40
35
30
25
20
15
10
5
0
0
25
50
75
100
Time of Night (%)

B10

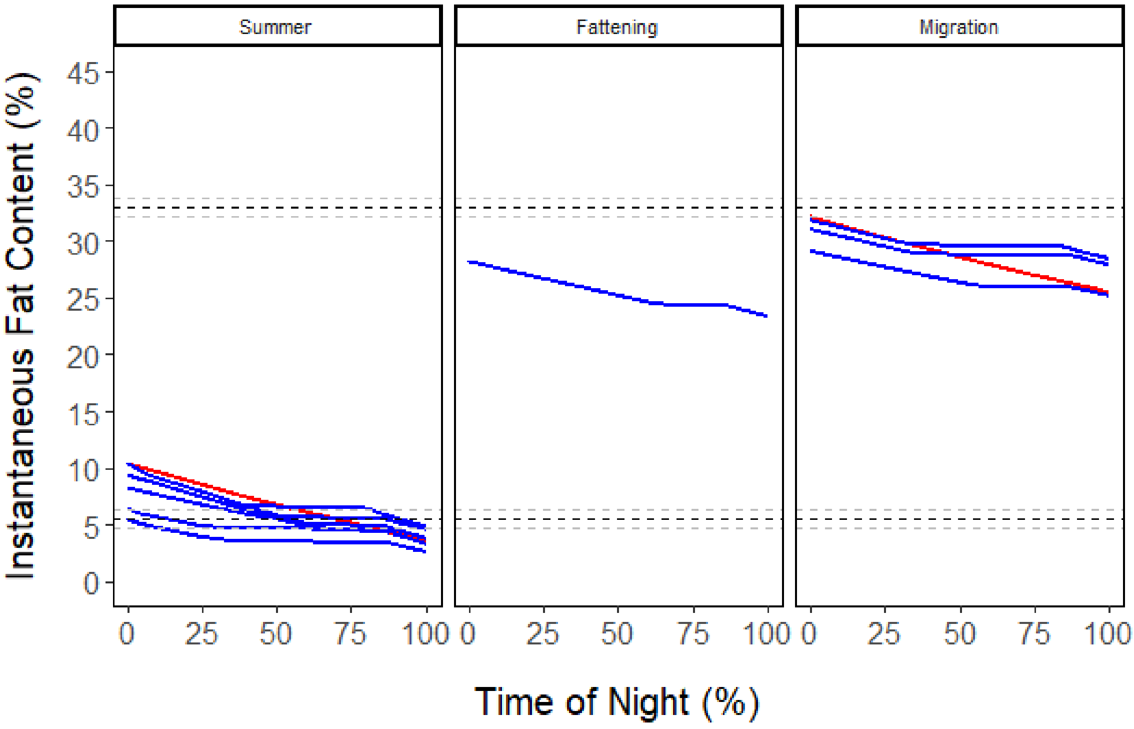
Summer
Fattening
Migration
Instantaneous Fat Content (%)
0
5
10
15
20
25
30
35
40
45
0
25
50
75
100
Time of Night (%)

B11

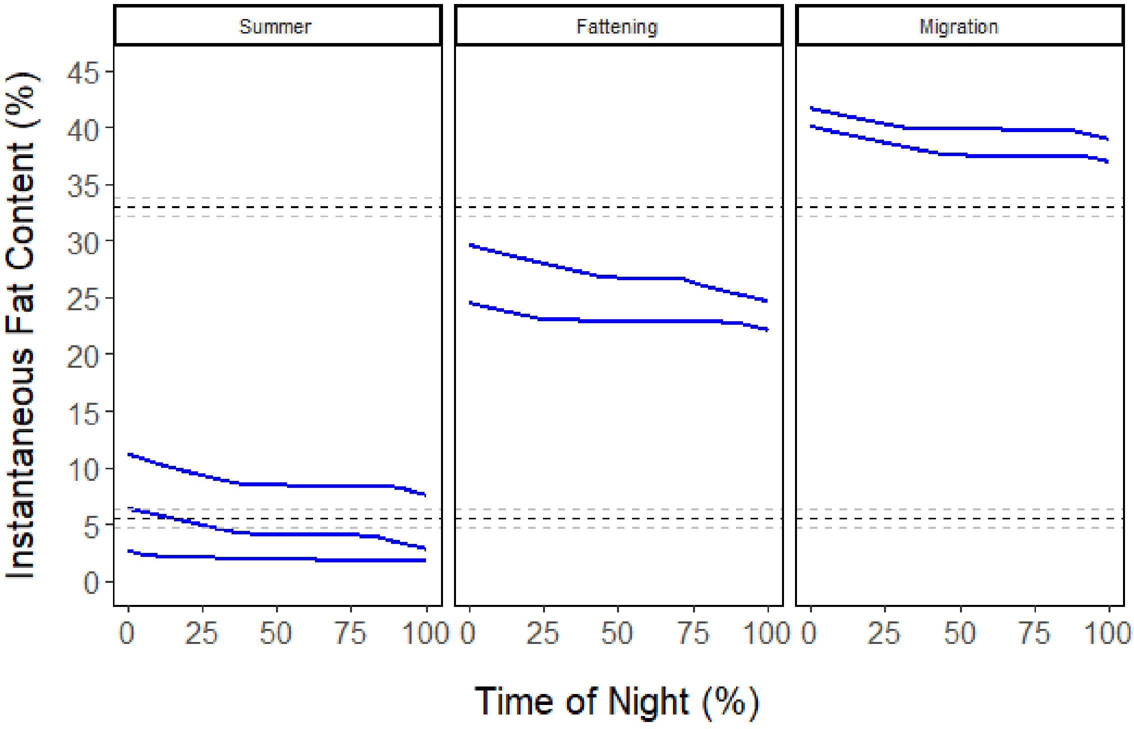
Summer
Fattening
Migration
Instantaneous Fat Content (%)
0
5
10
15
20
25
30
35
40
45
0
25
50
75
100
Time of Night (%)

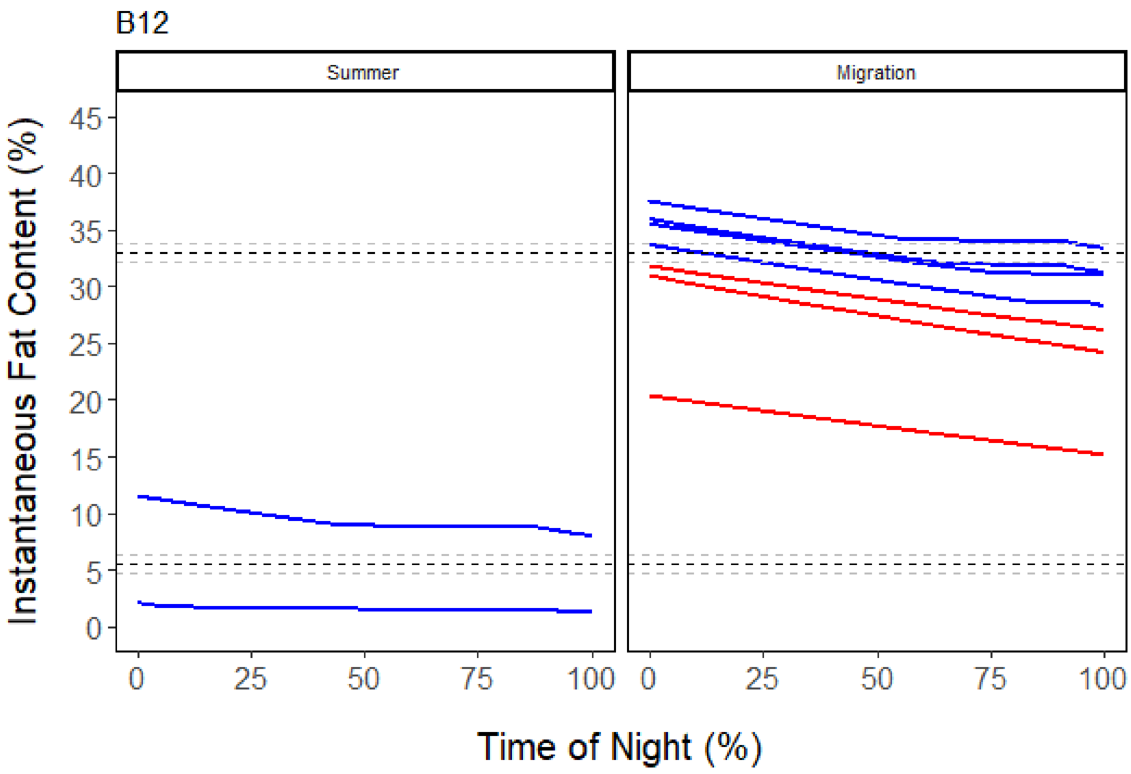
B12
Summer
Migration
Instantaneous Fat Content (%)
0
5
10
15
20
25
30
35
40
45
0
25
50
75
100
0
25
50
75
100
Time of Night (%)

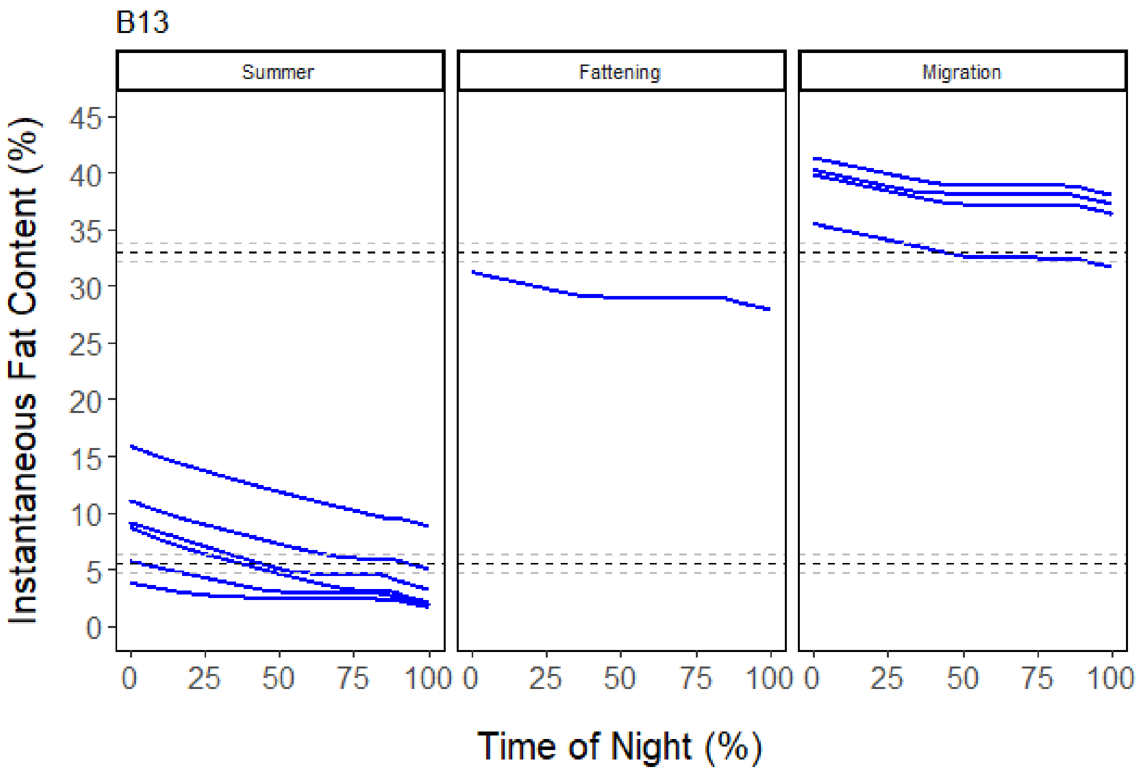
B13
Summer
Fattening
Migration
Instantaneous Fat Content (%)
0
5
10
15
20
25
30
35
40
45
0
25
50
75
100
0
25
50
75
100
0
25
50
75
100
Time of Night (%)

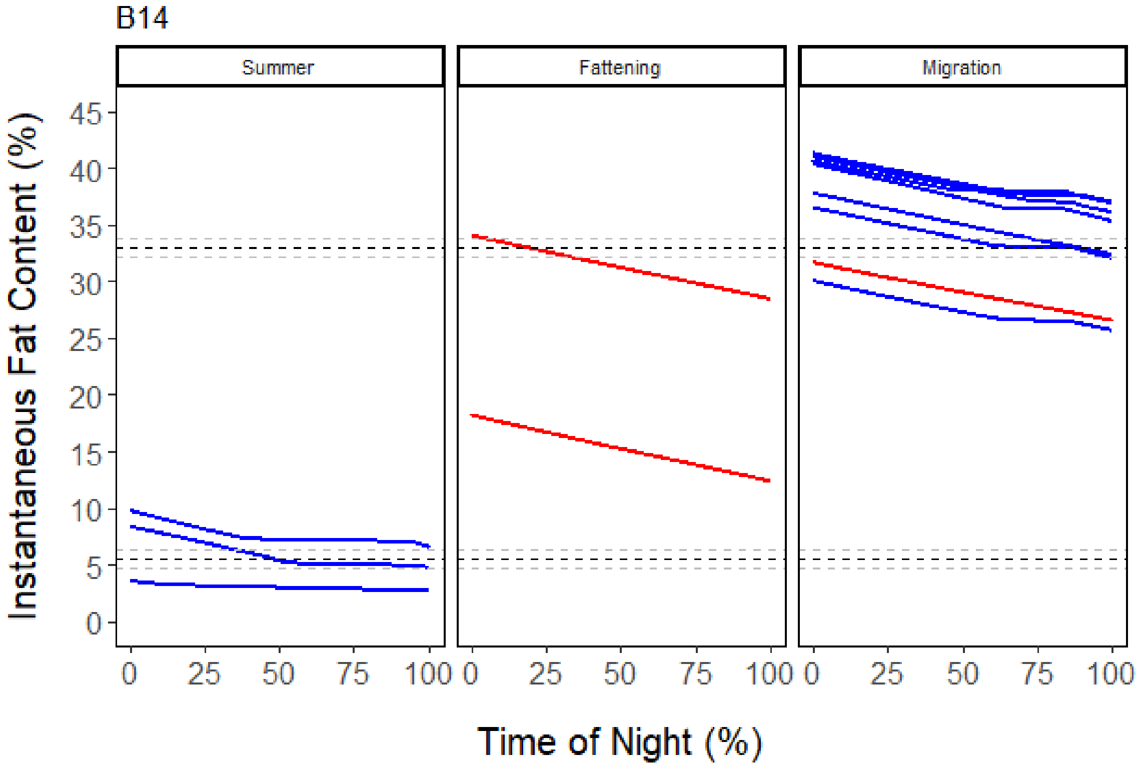
B14
Summer
Fattening
Migration
Instantaneous Fat Content (%)
0
5
10
15
20
25
30
35
40
45
0
25
50
75
100
Time of Night (%)

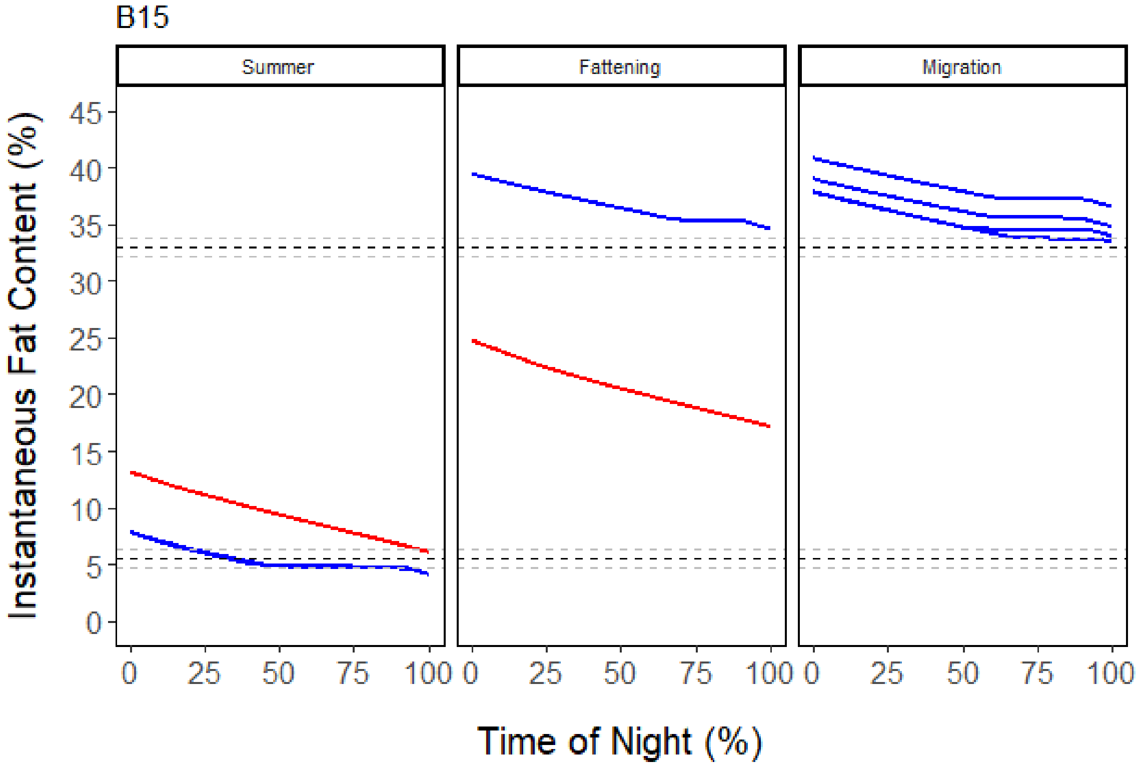
B15
Summer
Fattening
Migration
Instantaneous Fat Content (%)
0
5
10
15
20
25
30
35
40
45
0
25
50
75
100
Time of Night (%)

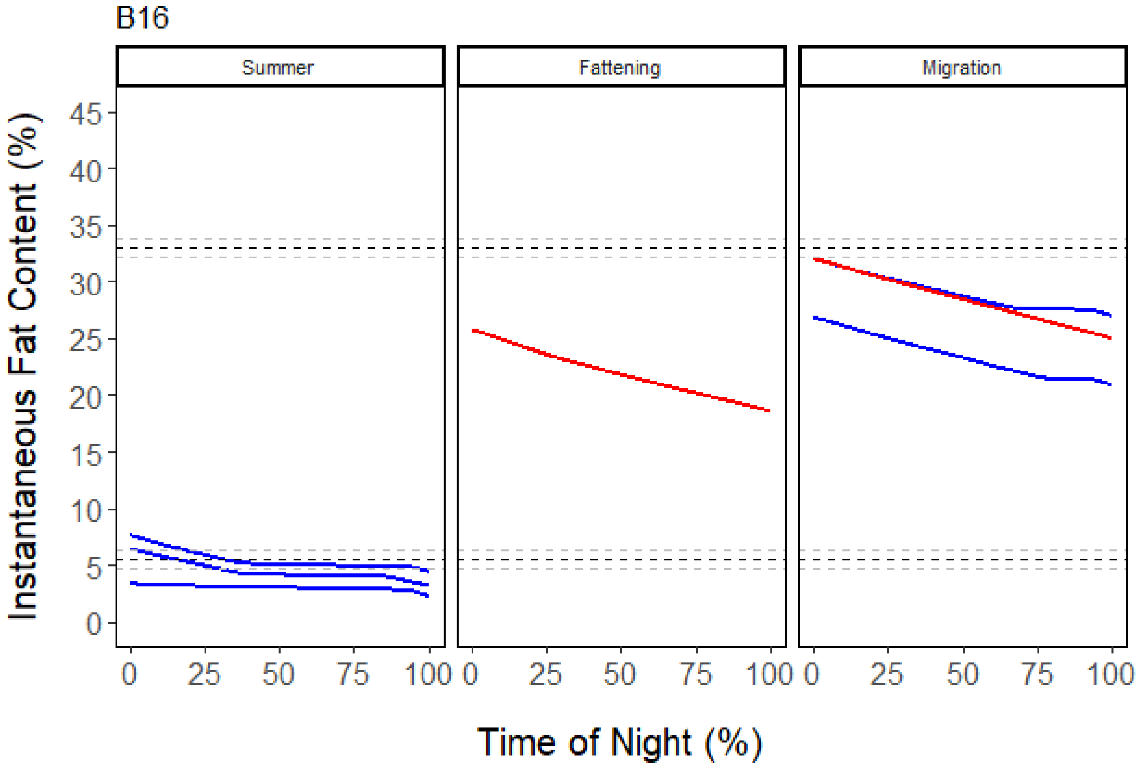
B16
Summer
Fattening
Migration
Instantaneous Fat Content (%)
0
5
10
15
20
25
30
35
40
45
0
25
50
75
100
Time of Night (%)
