# Supplementary material for: Reversal of the adipostat control of torpor during migration in hummingbirds
Source: eLife. 2021 Dec 6;10:e70062. doi: 10.7554/eLife.70062 (PMC8719877; doi:10.7554/eLife.70062)
Supplement: Supplementary file 3. — These data points were smoothed (greay line), and the slope of these points was used to define breeding, fattening, and migration periods for each bird, which are shaded blue, yellow, and red, respectively. Non-fatteners are also included and shaded dark red. The first panel shows night length throughout the study period. [file elife-70062-supp3.pdf]

Supplementary File 3. Morning body mass (black points) following focal nights across the entire study period, starting at the date of capture. These data points were smoothed (grey line), and the slope of these points was used to define breeding, fattening, and migration periods for each bird, which are shaded blue, yellow, and red, respectively. Non-fatteners are also included and shaded dark red. The first panel shows night length throughout the study period.

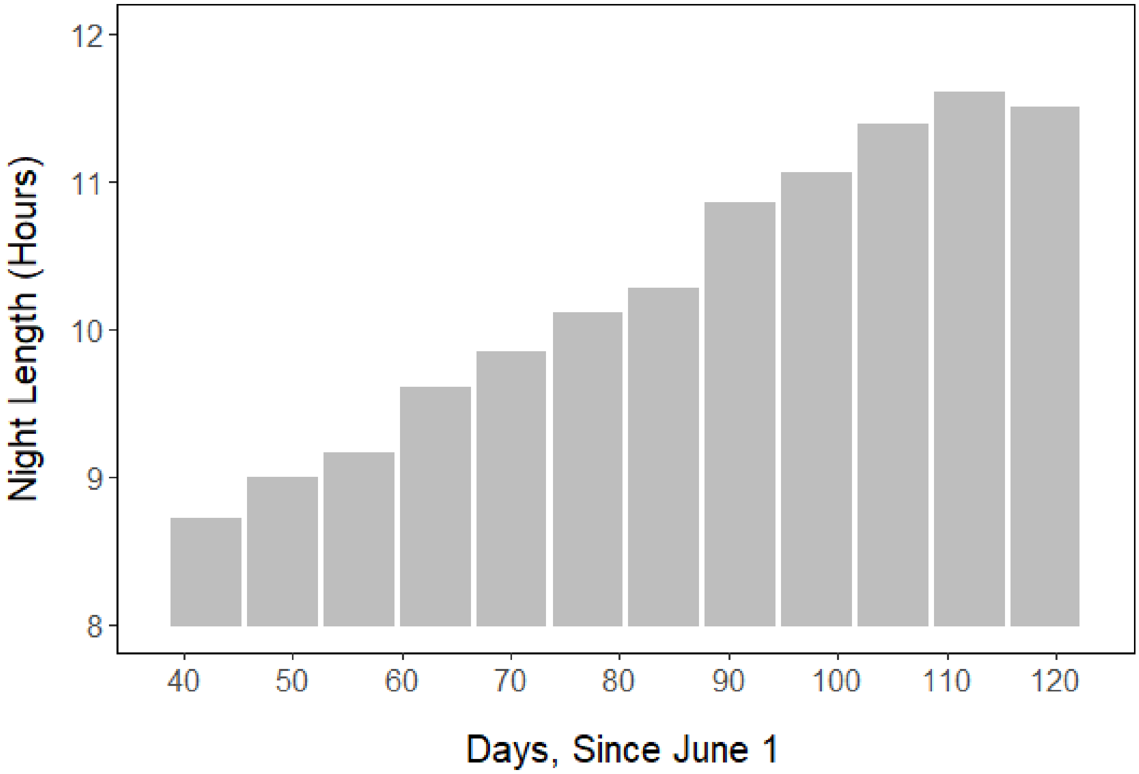

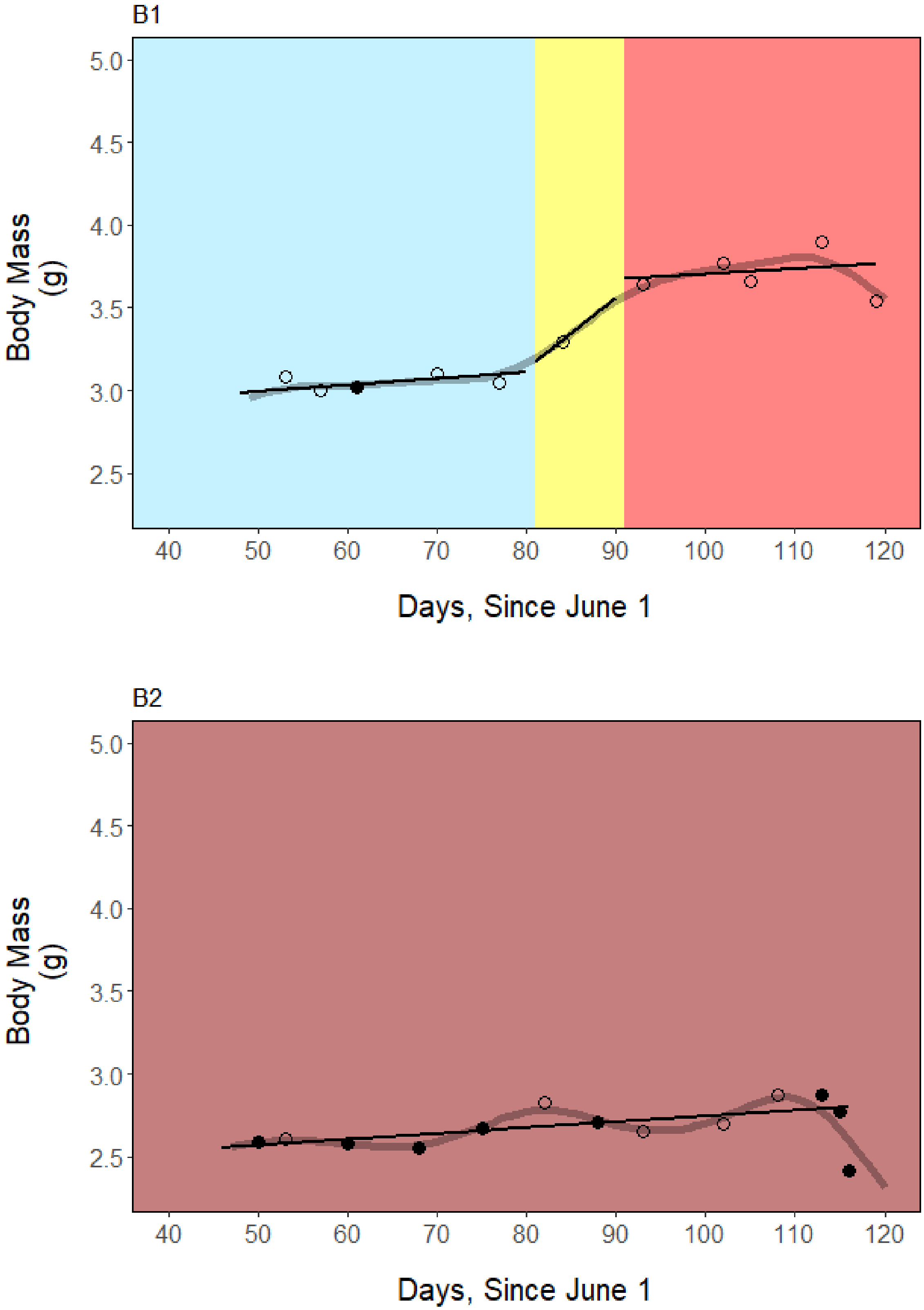
B1
Body Mass
(g)
5.0
4.5
4.0
3.5
3.0
2.5
40
50
60
70
80
90
100
110
120
Days, Since June 1
B2
Body Mass
(g)
5.0
4.5
4.0
3.5
3.0
2.5
40
50
60
70
80
90
100
110
120
Days, Since June 1

B3

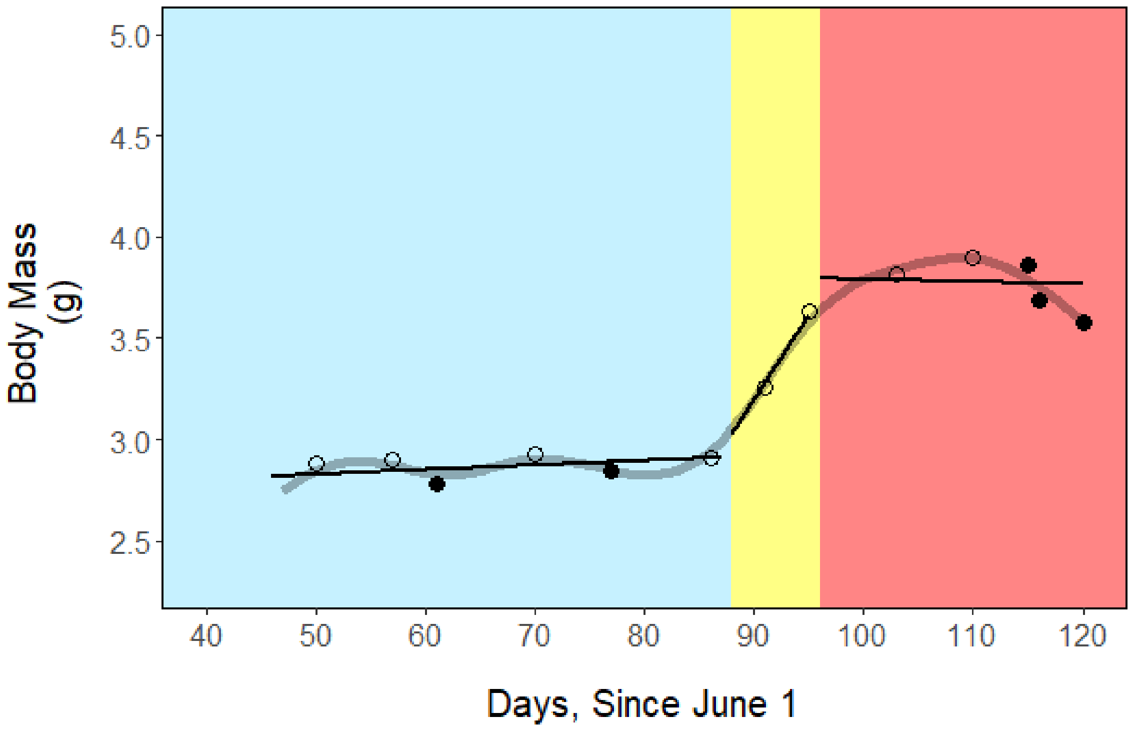

5.0
4.5
4.0
3.5
3.0
2.5
Body Mass (g)
40
50
60
70
80
90
100
110
120
Days, Since June 1

B4

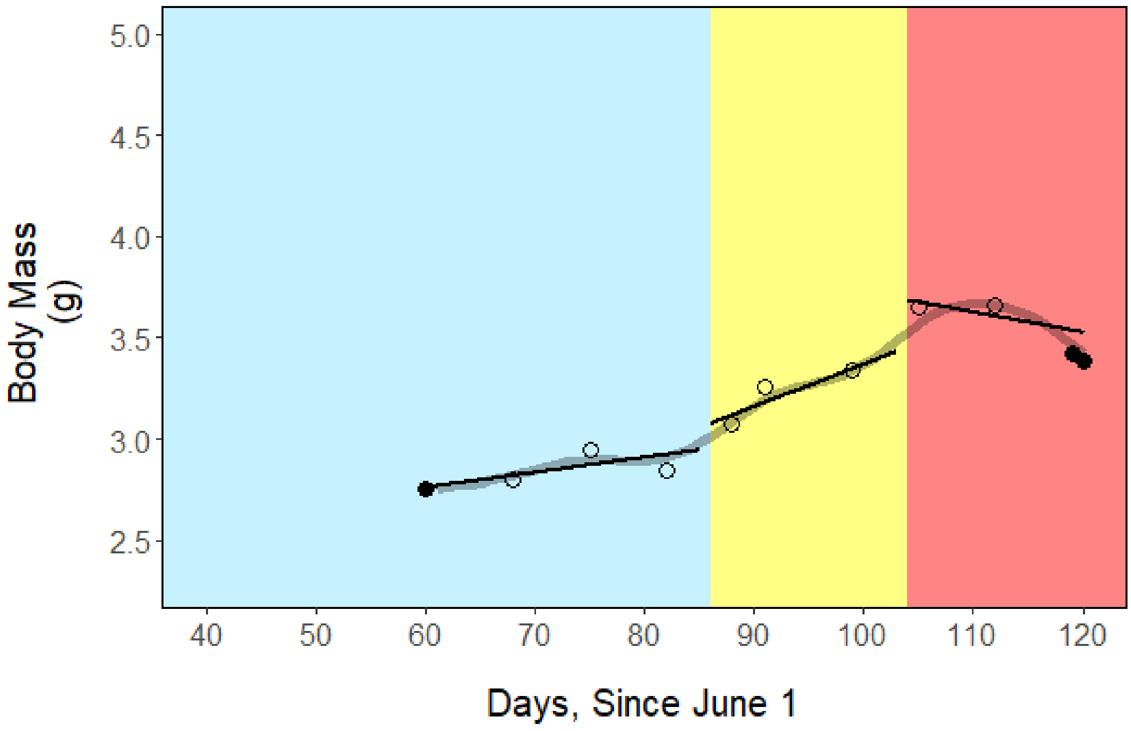

5.0
4.5
4.0
3.5
3.0
2.5
Body Mass (g)
40
50
60
70
80
90
100
110
120
Days, Since June 1

B5

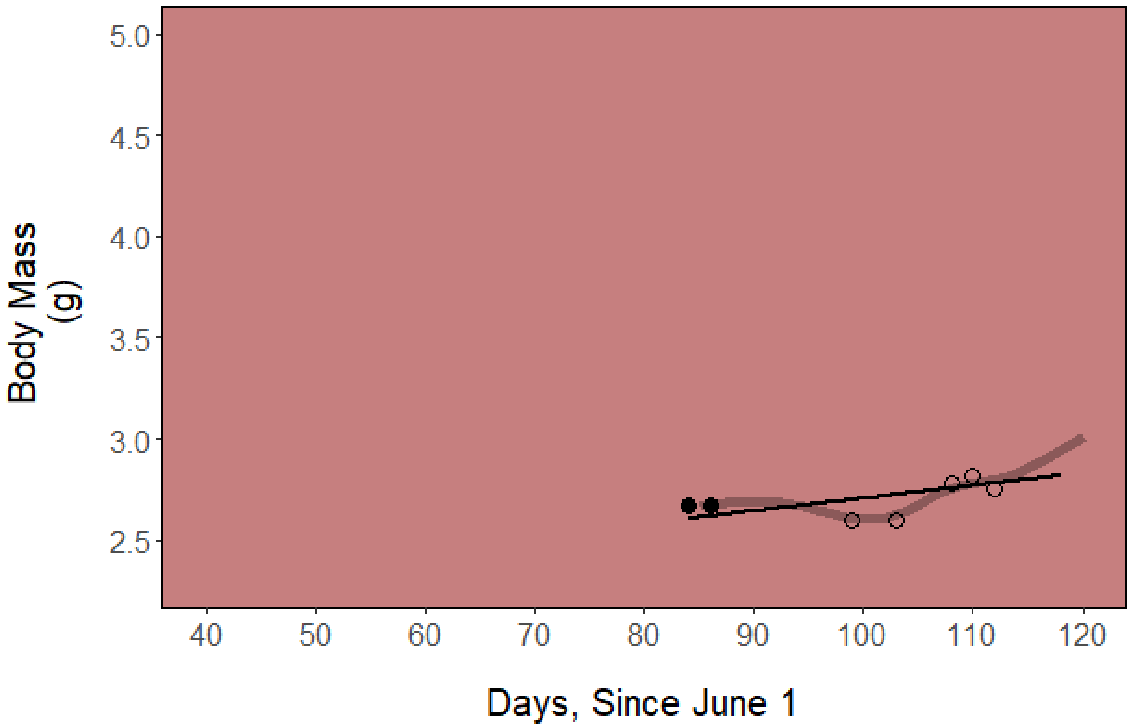

Body Mass
(g)
5.0
4.5
4.0
3.5
3.0
2.5
40
50
60
70
80
90
100
110
120
Days, Since June 1

B6

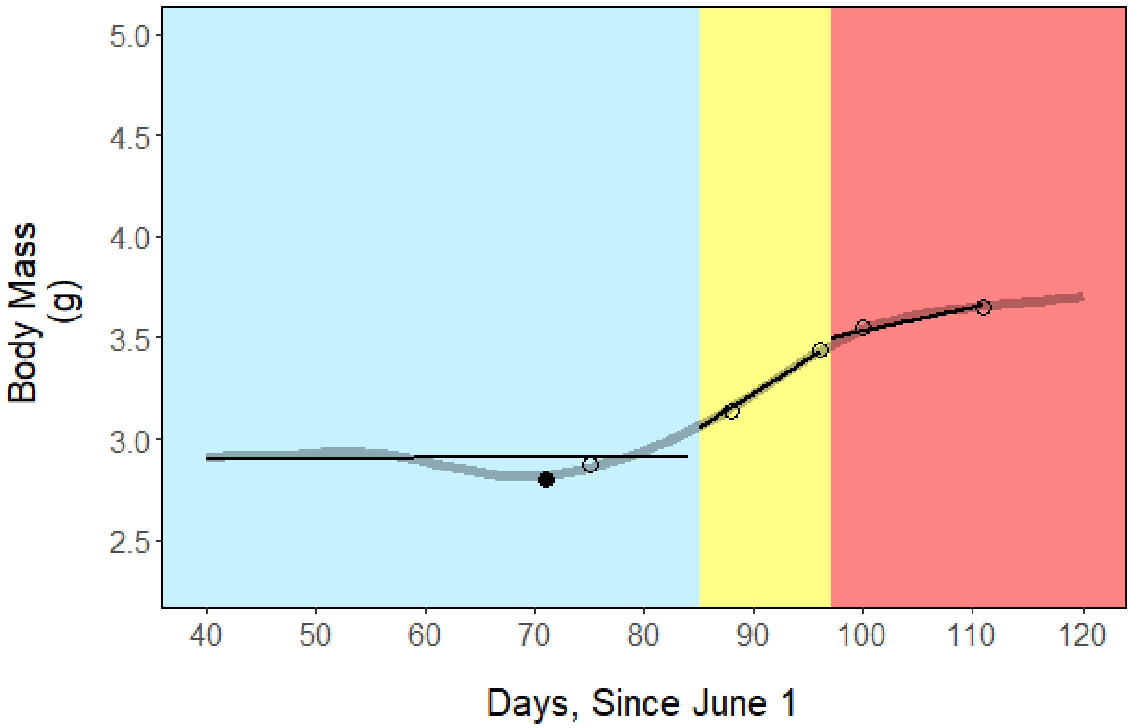

Body Mass
(g)
5.0
4.5
4.0
3.5
3.0
2.5
40
50
60
70
80
90
100
110
120
Days, Since June 1

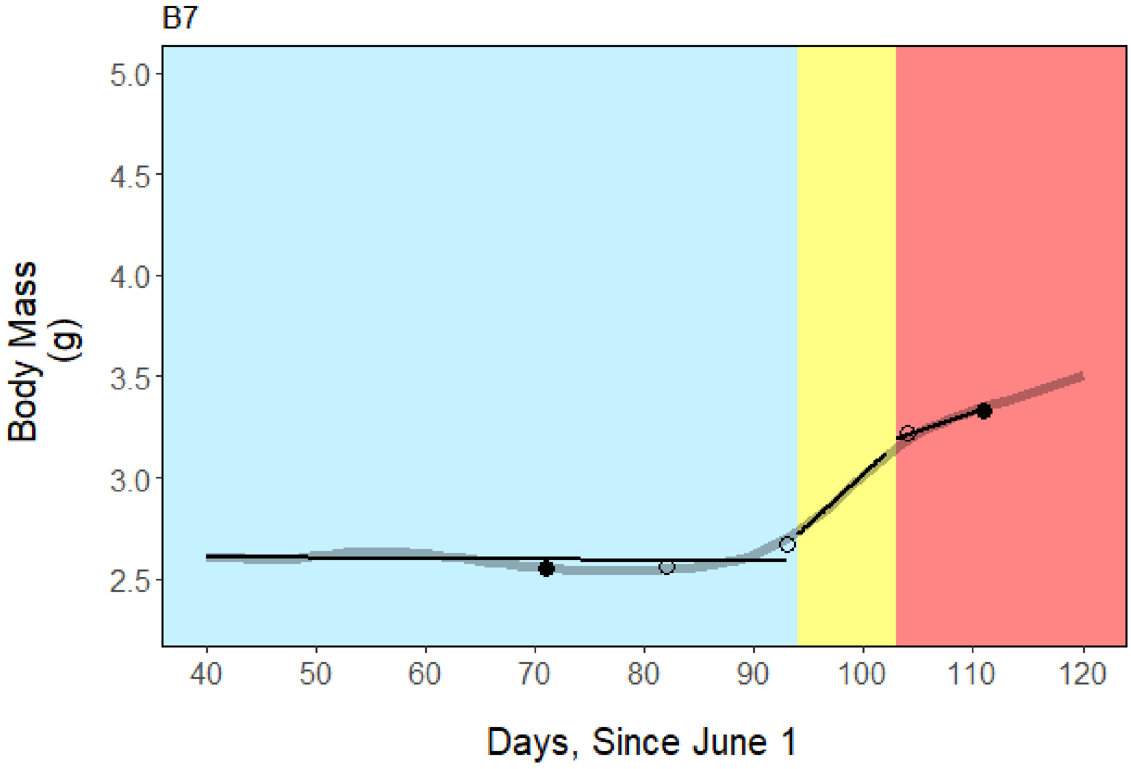
B7
Body Mass
(g)
5.0
4.5
4.0
3.5
3.0
2.5
40
50
60
70
80
90
100
110
120
Days, Since June 1

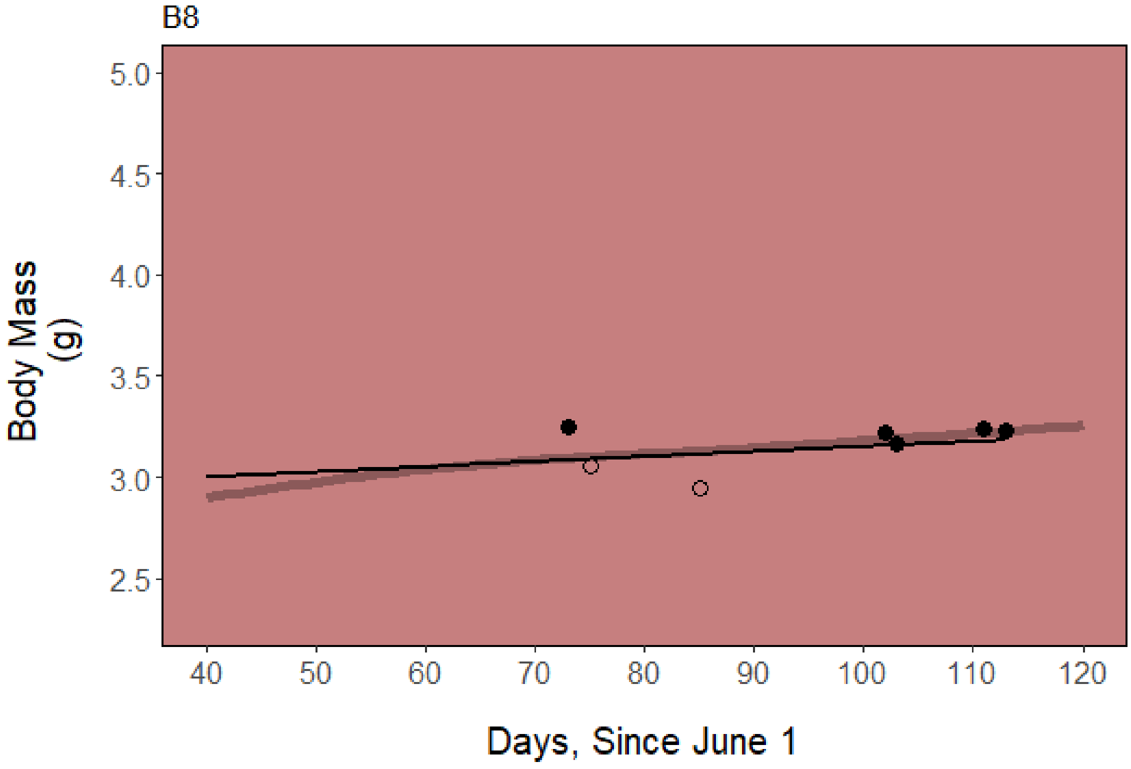
B8
Body Mass
(g)
5.0
4.5
4.0
3.5
3.0
2.5
40
50
60
70
80
90
100
110
120
Days, Since June 1

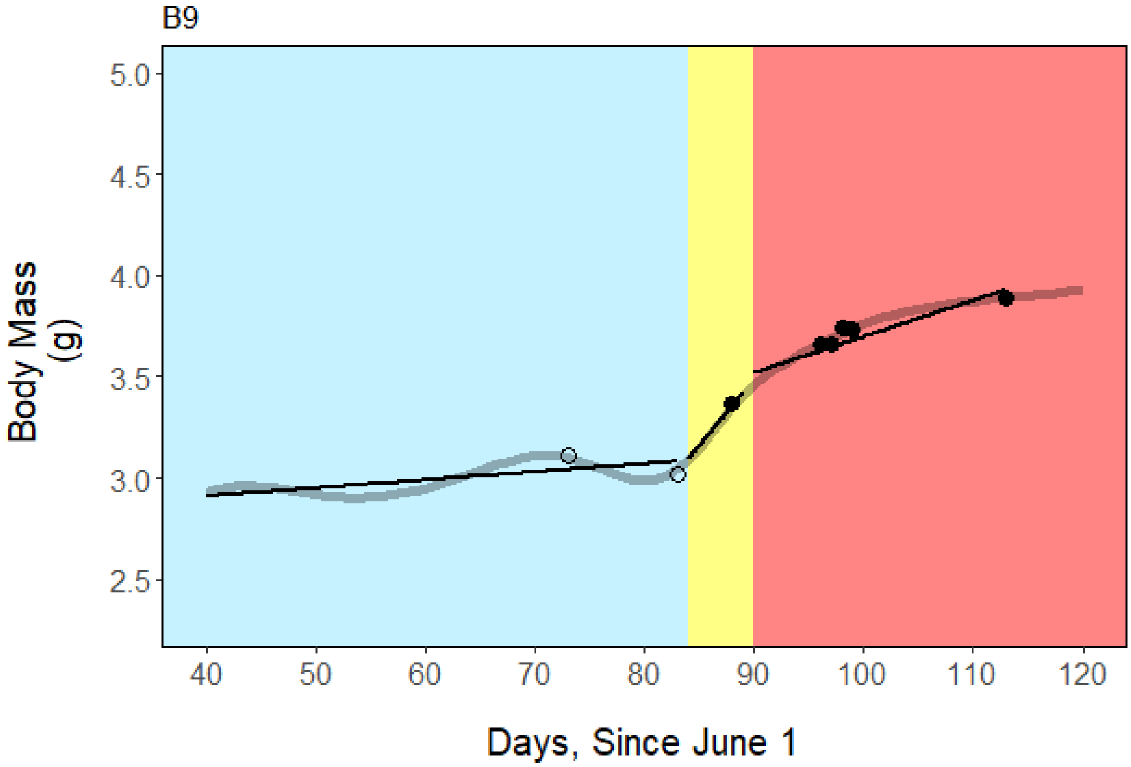
B9
Body Mass (g)
5.0
4.5
4.0
3.5
3.0
2.5
40
50
60
70
80
90
100
110
120
Days, Since June 1

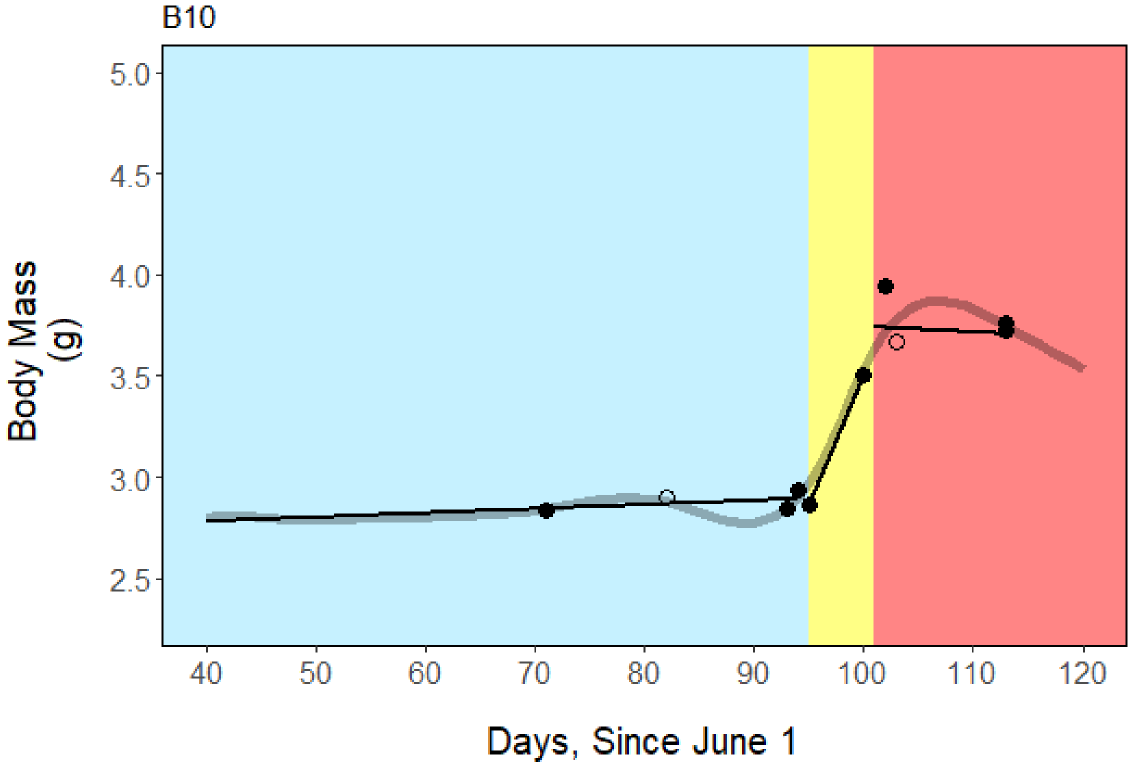
B10
Body Mass (g)
5.0
4.5
4.0
3.5
3.0
2.5
40
50
60
70
80
90
100
110
120
Days, Since June 1

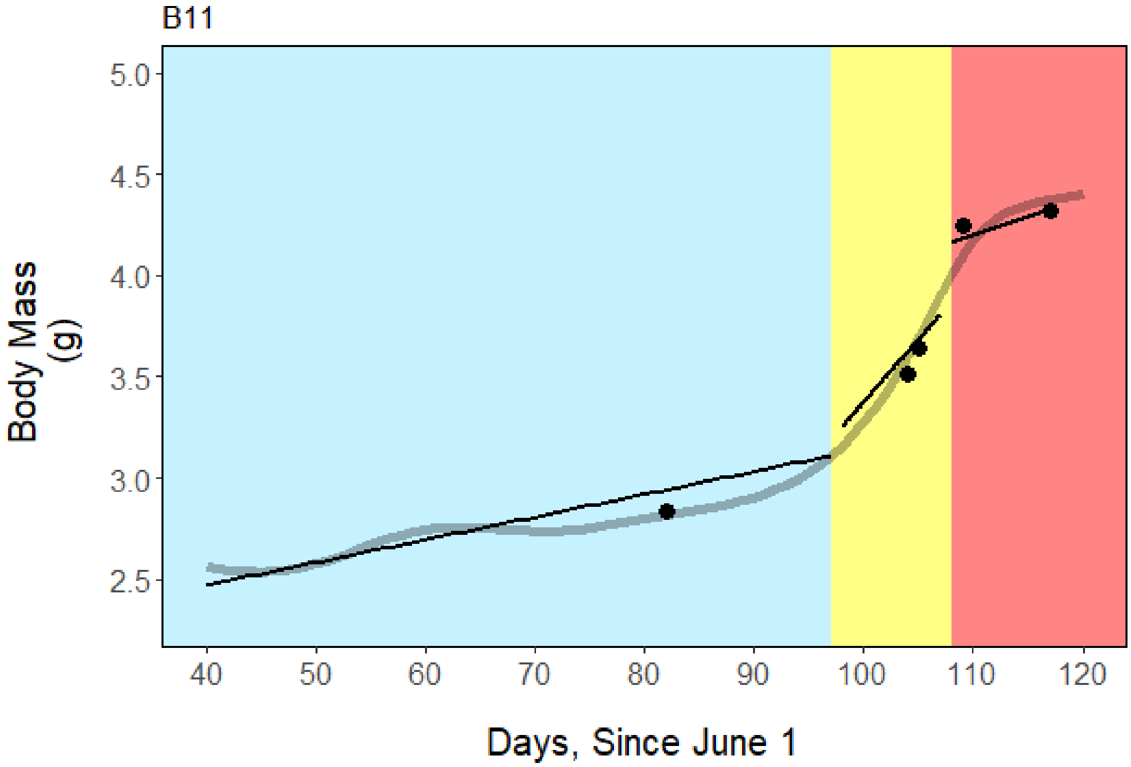
B11
Body Mass (g)
5.0
4.5
4.0
3.5
3.0
2.5
40
50
60
70
80
90
100
110
120
Days, Since June 1

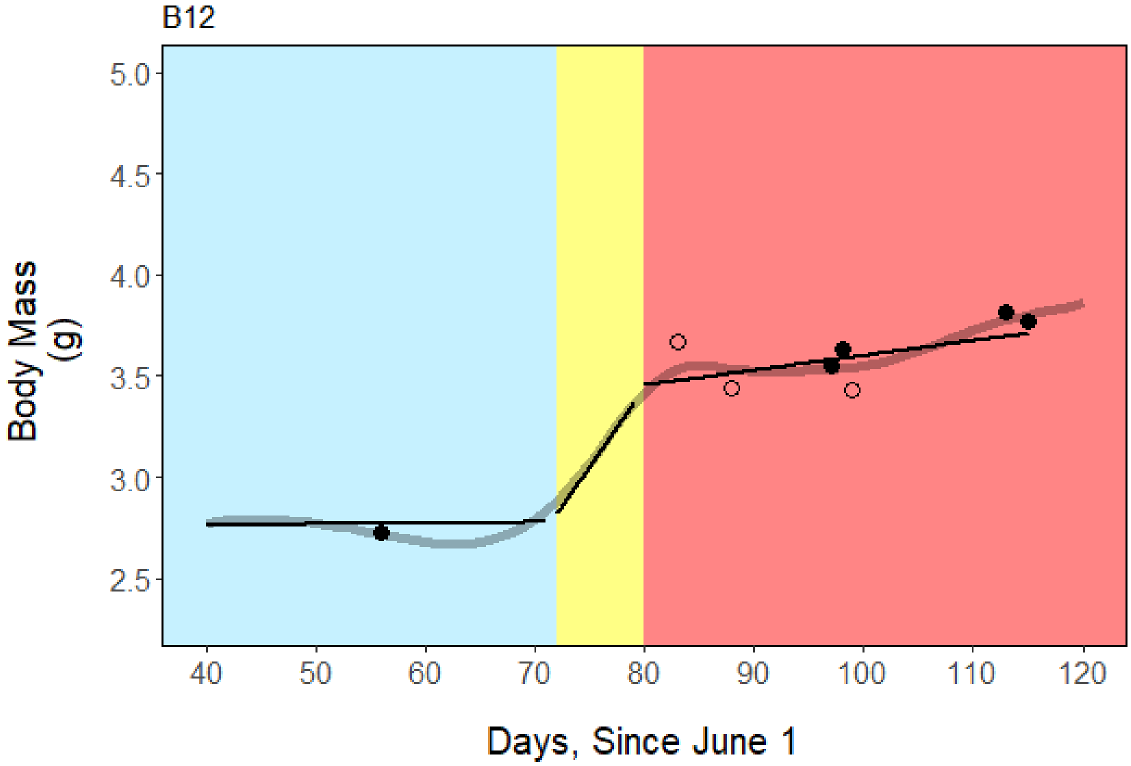
B12
Body Mass (g)
5.0
4.5
4.0
3.5
3.0
2.5
40
50
60
70
80
90
100
110
120
Days, Since June 1

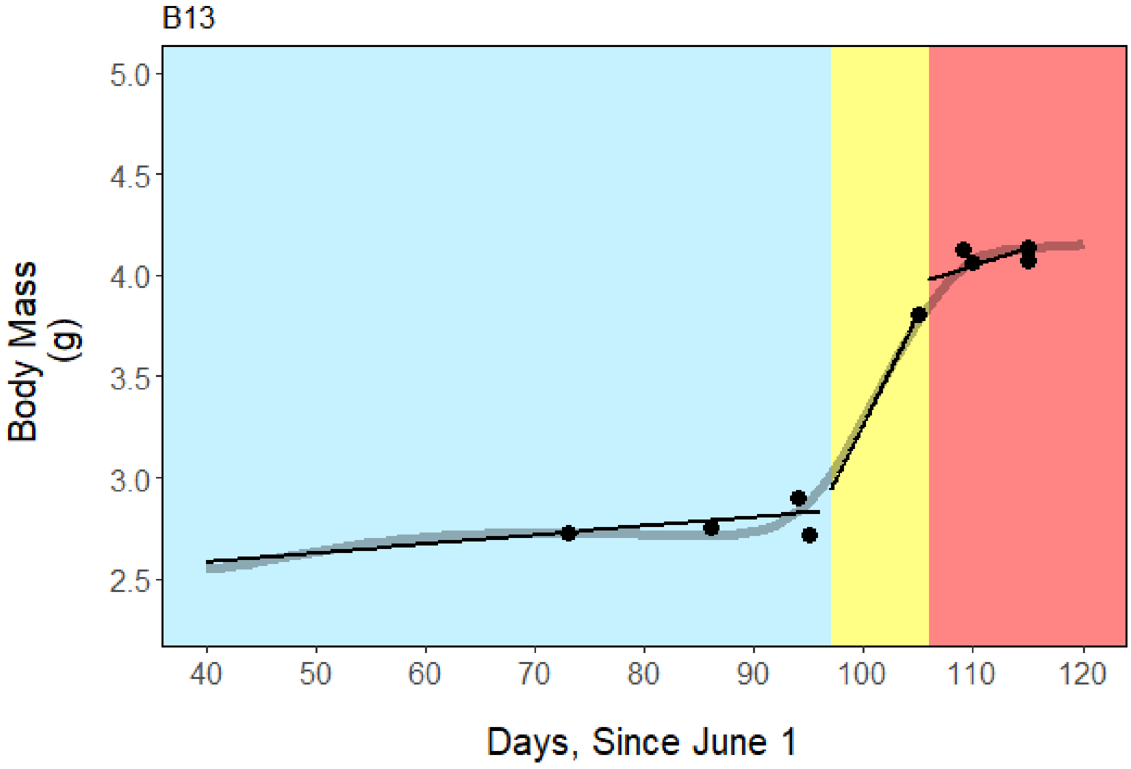
B13
Body Mass (g)
5.0
4.5
4.0
3.5
3.0
2.5
40
50
60
70
80
90
100
110
120
Days, Since June 1

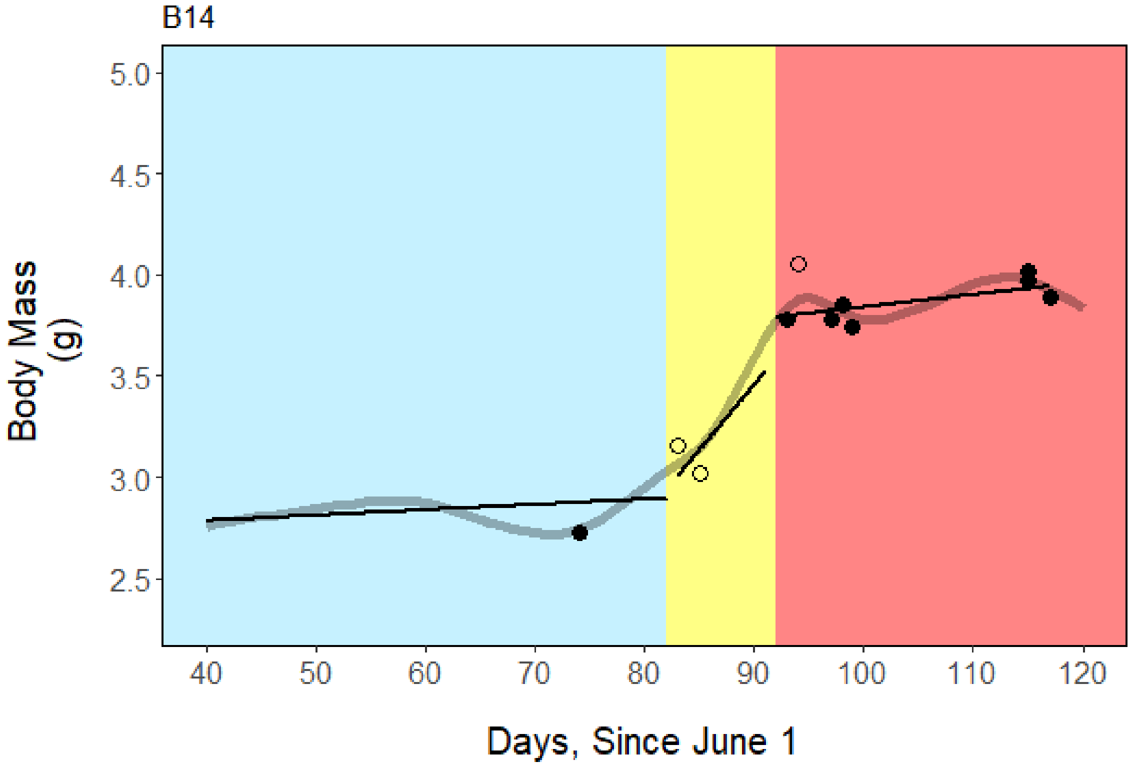
B14
Body Mass (g)
5.0
4.5
4.0
3.5
3.0
2.5
40
50
60
70
80
90
100
110
120
Days, Since June 1

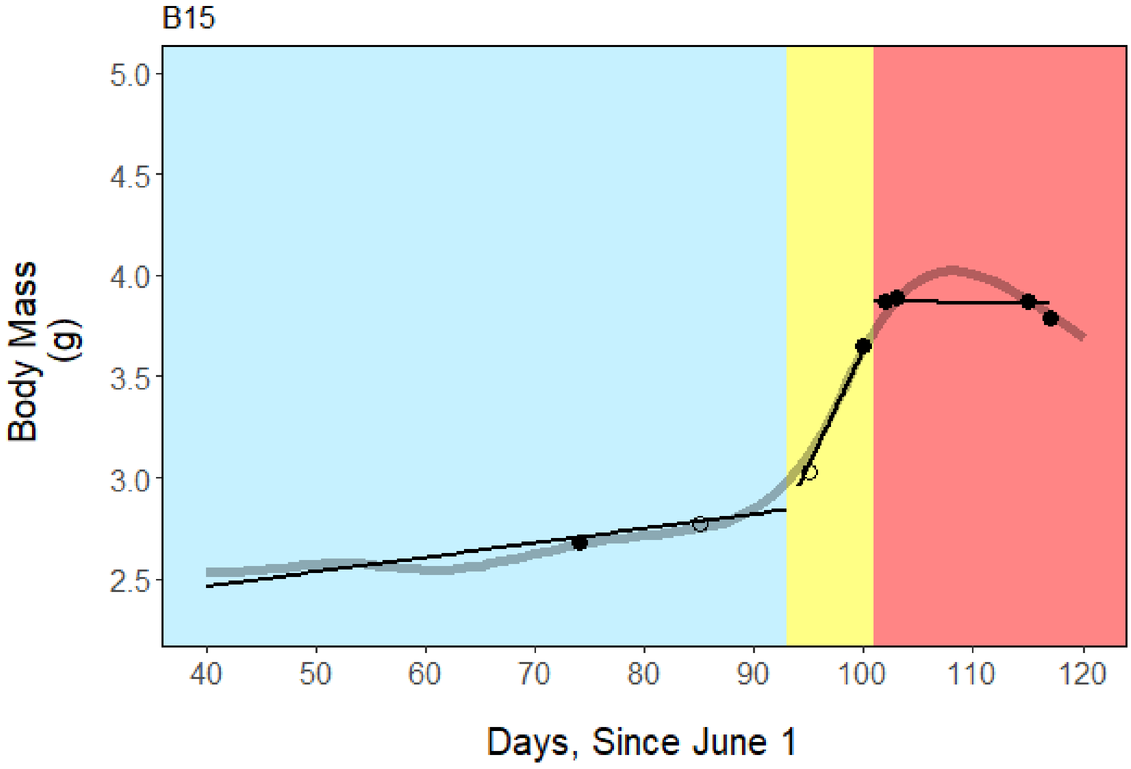
B15
Body Mass (g)
5.0
4.5
4.0
3.5
3.0
2.5
40
50
60
70
80
90
100
110
120
Days, Since June 1

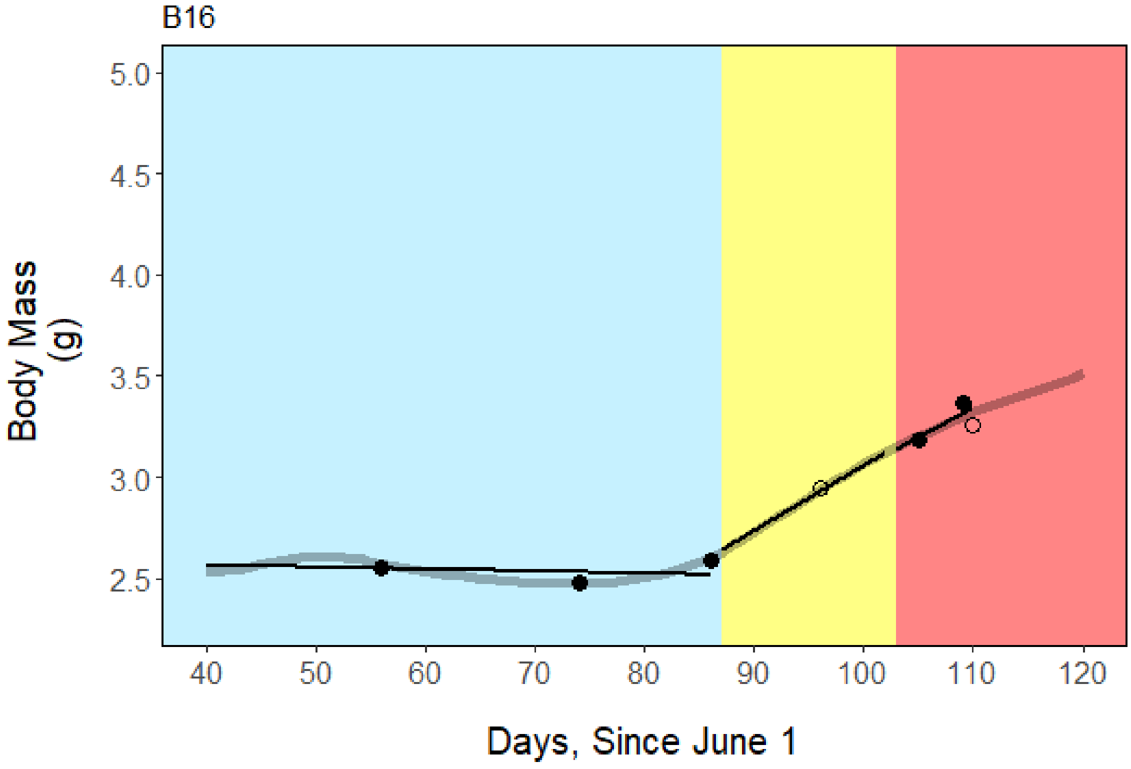
B16
Body Mass (g)
5.0
4.5
4.0
3.5
3.0
2.5
40
50
60
70
80
90
100
110
120
Days, Since June 1
